# Traditional supports and contemporary disrupters of high fertility desires in sub-Saharan Africa: a scoping review

**DOI:** 10.1186/s12978-023-01627-7

**Published:** 2023-06-06

**Authors:** Anna C. Church, Mobolaji Ibitoye, Shibani Chettri, John B. Casterline

**Affiliations:** 1 Department of Sociology, The Ohio State University, 238 Townshend Hall, 1885 Neil Avenue Mall, Columbus, OH 43210 USA; 2 Institute for Population Research, The Ohio State University, 060 Townshend Hall, 1885 Neil Avenue Mall, Columbus, OH 43210 USA; 3 College of Public Health, The Ohio State University, 250 Cunz Hall, 1841 Neil Ave, Columbus, OH 43210 USA

**Keywords:** Sub-Saharan Africa, Fertility desires, Scoping review, Men and women, High fertility

## Abstract

**Rationale:**

The desired number of children is markedly higher in Sub-Saharan Africa (SSA) than in other major regions. Efforts to understand how and why these desires are generated and maintained have yielded a broad research literature. Yet there is no full picture of the range of contextual, cultural, and economic factors that support and disrupt high fertility desires.

**Objective:**

This scoping review synthesizes thirty years of research on the determinants of fertility desires in SSA to better understand what factors underlie men and women’s stated fertility desires and how they weigh the costs and benefits of having (more) children.

**Method:**

We identified and screened 9863 studies published from 1990 to 2021 from 18 social science, demographic, and health databases. We appraised determinants of fertility desires from 258 studies that met inclusion criteria according to their roles as traditional supports or contemporary disrupters of high fertility desires.

**Results:**

We identified 31 determinants of high fertility desires, which we organized into six overarching themes: economy and costs; marriage; the influence of others; education and status; health and mortality; and demographic predictors. For each theme, we summarize ways in which the determinants both support and disrupt high fertility desires. We find that high fertility remains desirable in many regions of sub-Saharan Africa but contemporary disrupters, such as the economic situations and increases to family planning and education, cause individuals to decrease their desired fertility with such decreases often viewed as a temporary adjustment to temporary conditions. Most included studies were quantitative, cross-sectional, and based on survey data.

**Conclusion:**

This review demonstrates how traditionally supportive and contemporary disruptive forces simultaneously influence fertility desires in sub-Saharan Africa. Future studies analyzing fertility desires in sub-Saharan Africa should be informed by the lived experiences of men and women in this region, with qualitative and longitudinal studies prioritized.

**Supplementary Information:**

The online version contains supplementary material available at 10.1186/s12978-023-01627-7.

## Plain language summary

In sub-Saharan Africa, both men and women continue to desire large numbers of children in contrast to most regions of the world where the desired number of children is near or below replacement level (around 2.0 children per couple). We conducted a comprehensive review of the existing research on the sources and drivers of the persistently high desired fertility in sub-Saharan Africa. Based on the review, 258 studies were included in this analysis. Several factors that influence fertility desires among men and women in this population were identified. We categorized them as either traditional supports (factors that have historically supported and promoted high fertility desires) or contemporary disrupters (factors that have more recently inhibited or discouraged high fertility desires). Although fertility desires are shaped by a wide range of factors that vary based on specific country and population, several overarching conclusions were clear. Contemporary changes in the economy and family have caused individuals to shift their fertility desires downward, and this shift is often viewed as a temporary adjustment to temporary conditions. Increased autonomy and formal schooling of women and availability of family planning can help position women to implement lower fertility desires. There is a need for more longitudinal research in this region to better understand how fertility desires may change over a person’s life, and for more qualitative studies that allow people to describe and explain their lives and fertility desires more directly in ways that allow for uncertainty and ambiguity.

**Supplementary Information:**

The online version contains supplementary material available at 10.1186/s12978-023-01627-7.

## Introduction

Sub-Saharan Africa (SSA) is the major region where fertility rates remain high [1]. Survey data indicate that most of this childbearing is desired. Fertility desires are higher in SSA than other major regions; the mean desired family size in SSA is 5.0 as compared to 2.9 in other low- and middle-income countries (LMICs) [2]. The higher fertility rates on average in SSA are a function of the relatively high desired fertility [3, 4].

For decades, social and health scientists, among others, have investigated the drivers and motivators behind high fertility desires in SSA. Most of this research has consisted of analysis of survey data, primarily Demographic and Health Surveys (DHS). This has been complemented by a smaller number of studies employing non-survey approaches, including qualitative interviews and ethnography. The pertinent research literature is diverse methodologically, with clear variation within and across countries and regions in SSA. Hence, no small set of specific studies provides a full picture of the range of contextual, cultural, and economic factors that drive fertility desires in SSA.

The goal of this scoping review is to synthesize research evidence from the last thirty years on the determinants of fertility desires in SSA. Specific research questions include: (1) What factors underlie men and women’s stated fertility desires? and (2) How do men and women weigh the costs and benefits of having (more) children? We consider how the existing literature has addressed these questions and provide a more expansive understanding of the influences of fertility desires in SSA. We also highlight gaps that future research should address. The theoretical framework guiding this review makes a basic distinction between traditional supports for high desired fertility and factors that disrupt these traditional supports, thereby prompting revised evaluations of the benefits and costs of childbearing.

### Theoretical framework

In the 1970s and early 1980s a prevailing view among scholars was that the high desired fertility in SSA, evident in both survey data and other types of empirical investigations, reflected the dominant social and cultural systems characterizing the region. Shaped by these systems, high fertility was rewarded, contributing to the normalization of high fertility desires and large family sizes. High fertility was perceived as important for the perpetuation of individual lineages, enlarging kinship groups and the survival of the extended family that was crucial for livelihoods (e.g. access to land) and other privileges dependent on personal connections [5–7]. Continuing a family’s line of descent and fulfilling responsibilities to communities and kin were important motivators of the desire for many children. Thus, the interests of the community and the extended kinship group weighed heavily on individuals’ reproductive intentions and behaviors [8]. Given the high importance attached to posterity, scholars surmised that lineage-based systems would offer considerable resistance to reductions in desired fertility (and, concomitantly, the likely returns on family planning programs) [9].

An alternative view was that the high fertility desires in SSA were not principally a function of social and cultural systems but, rather, a rational response to the continent’s specific history with colonialism [10]. Faced with extreme and prolonged high-mortality conditions during colonization, reproductive regimes in African societies became efficient at maximizing fertility and, to the extent feasible, child survival, while colonizers also promoted fertility maximization to supply a steady stream of workers [10]. Over several generations, these historically-grounded factors generated high-fertility norms that became resistant to change [11]. Even though infant and child mortality rates have fallen since independence, especially since 1990, fertility desires (and the resulting fertility rates) have been slow to follow.

Theoretical frameworks for fertility transition that have enjoyed some success in explaining fertility declines since 1950 in other regions have proven inadequate in explaining the slow, and sometimes non-existent, progression of fertility decline in SSA [1, 12, 13]. With this realization, it has become common for scholars to speak of “African exceptionalism” [2, 14]. We offer no judgment about the course of reproductive change in SSA that marks it off as “exceptional.” Instead, we note that fertility decline is underway throughout SSA, albeit at widely varying pace, corresponding with survey evidence of declines in desired fertility. This is *prima facie* evidence that the forces of the aforementioned traditional and historical influences that shaped fertility desires in SSA over many generations are now challenged by new societal developments that have disrupted these longstanding influences.

What demographic and socioeconomic changes are acting to drive down desired fertility? Many of the candidates are readily recognized and have been the subject of extensive empirical investigation. These include changes in the economy, increasing importance and cost of children’s education [15], sharp reductions in infant and child mortality [16], the introduction of family planning programs accompanied by mass media campaigns to distribute contraceptive awareness [17], and more recently climate change that has affected agricultural production and land usage [18]. The extent to which these contemporary and traditional factors are changing and continuing to influence individuals’ fertility desires in SSA is less understood.

With this scoping review, we aim to provide a more comprehensive understanding of the full array of influences on fertility desires in SSA. Our review of the immense literature during the past thirty years is organized around two major theoretical themes:   (1) traditional supports of high fertility desires and (2) contemporary disruptors.

## Methods

We conducted this scoping review guided by the framework proposed by Arskey and O'Malley [19] and further elaborated by Levac, Colquhoun, and Brien [20] and Daudt, van Mossel, and Scott [21] We address two of the purposes of scoping reviews specified by Arskey and O’Malley [19]. First, we summarize the extant research on the determinants of fertility desires in sub-Saharan Africa. Second, we identify gaps in the existing literature. At the outset, we developed a protocol to guide the conduct of the scoping review, adhering to the format outlined in the International Prospective Register of Systematic Reviews (PROSPERO) [22]. However, we did not register the protocol because PROSPERO does not currently register scoping review protocols. We report the scoping review process and findings in accordance with established reporting guidelines (Additional file 1: Appendix A, Table S1) [23].

### Literature search strategy

With the help of two experienced research librarians, we developed, pilot tested, and refined a search strategy pertaining to the population (men and women of reproductive age), concept (fertility desires), and context of interest (SSA). We used the search strategy to identify relevant social science, demographic, and health literature published from 1990 to 2021 in 18 databases (Academic Search Complete, Africa-wide, CAB Abstracts and Global Health, CINAHL, Embase, Gender Watch, ProQuest [Dissertations and Theses Global, International Bibliography of the Social Sciences, PAIS Index, Sociological Abstracts, Social Science Database, Sociology Database], PsycINFO, PubMed, Scopus, Social Sciences Abstracts, SocINDEX, and Web of Science Core Collection). We searched each database using free-text keywords and controlled vocabulary specific to the database. We ran the initial search in July 2020 and updated it to include more recent literature in April 2021. We include a sample search strategy used for the PubMed database in Additional file 1: Appendix B, Table S2.

### Inclusion and exclusion criteria

#### Participants

We include studies conducted with men and/or women of reproductive age (15–49 years old). This limits the review to studies conducted among men and women for whom the topic of interest is most salient. We excluded studies that focused exclusively on special populations defined by specific health conditions that may affect their fertility intentions (e.g., HIV positive people).

#### Concept

We included studies if they assessed or discussed factors that affect participants’ fertility desires, excluding studies focused on family planning or contraception that did not specifically address determinants of fertility desires. Due to the wide range of disciplinary fields included, fertility desires were operationalized in several ways across studies. Table 1 presents the various ways in which fertility desires were measured in the included studies.

**Table 1:** Key terms for measuring fertility desires

Term	Definition
Desire for a/another child	Desire for a child in the future or desire for an additional child if the person already has at least one living child
	Can be further broken down by temporality—if a person wants a/another child soon, later, or are undecided as to when
Ideal/preferred/desired family size	The family size wanted in one’s lifetime
Ideal/desired number of children	The number of children wanted in one’s lifetime
Desire for no more children	Do not want any children at all or no additional children if the person already has at least one living child

#### Context

The review covers studies conducted in SSA from 1990 to 2021. The 1990 cut-off date was chosen because the DHS Program, the basis of most literature on fertility desires in SSA, was launched in Africa in 1986, with the first set of articles from the data being published in the early 1990s.

#### Types of studies

We included all study types—quantitative, qualitative, and mixed methods—in the review, and incorporated a diversity of publication types including journal articles, book chapters, conference papers, dissertations, working papers, and research reports.

### Literature selection process

**Fig. 1:**
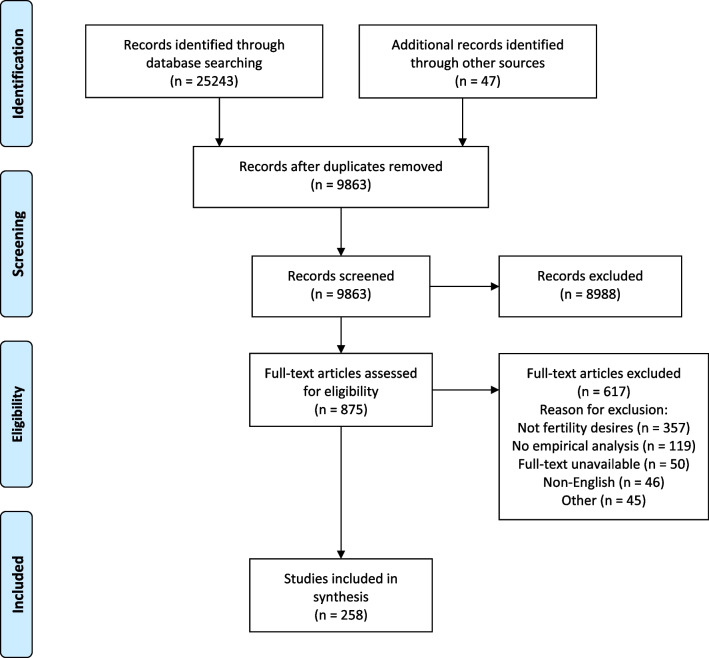
PRISMA flowchart

We used the citation manager Zotero to deduplicate all search results. We then used the online systematic review software Covidence for screening and data extraction of the remaining search results. Two reviewers independently screened each search result. First all titles and abstracts were screened for relevance. Next, the full texts of all relevant results were assessed against the inclusion and exclusion criteria. We resolved disagreements at each stage through discussion until consensus was reached. When the two reviewers could not come to an agreement, a third reviewer helped resolve disagreements. This process yielded 258 included studies. Figure 1 shows how many results were eliminated in each stage and the reasons for exclusion.

### Data synthesis

We extracted data from all studies that met the inclusion criteria using a standardized form developed specifically for the review. We pilot tested the form on a selection of ten studies and refined it. Two reviewers then independently extracted data from all the included studies using the finalized form. Extracted data included study characteristics, sample characteristics, operationalization of fertility desires, and study findings on fertility desires. Findings from the studies were coded and narratively synthesized according to identified determinants of fertility desires (see Table 2). Full citation information for all articles included in the review is available in the Additional file 1: Appendix C, Table S3. Data extracted from each included article is available upon request from the authors.

**Table 2:** Frequency of determinants

Traditional/historical support	Frequency	Contemporary disrupter	Frequency
Age	96	Education	130
Religion	84	Economy/costs	69
Place of residence	81	Socioeconomic status	68
Polygyny	74	Family planning	60
Parity	70	Employment	44
Child/infant mortality	60	Status of women	34
Sex preference	60	Spousal joint decision-making	29
Children as economic resource	57	HIV/AIDS epidemic	20
Security in old age	55	Mass media	19
Value of children	54	Women’s health concerns	9
Community influence	48	Environmental factors	8
Marital stability	47	Conflict/civil unrest/violence	6
Sex of participant	43	Migration	4
Lineage/clan influence	30	Legacy of colonialism/modernity	2
Family influence	29		
Ethnicity	23		
Spouse influence	22		

## Results

**Fig. 2:**
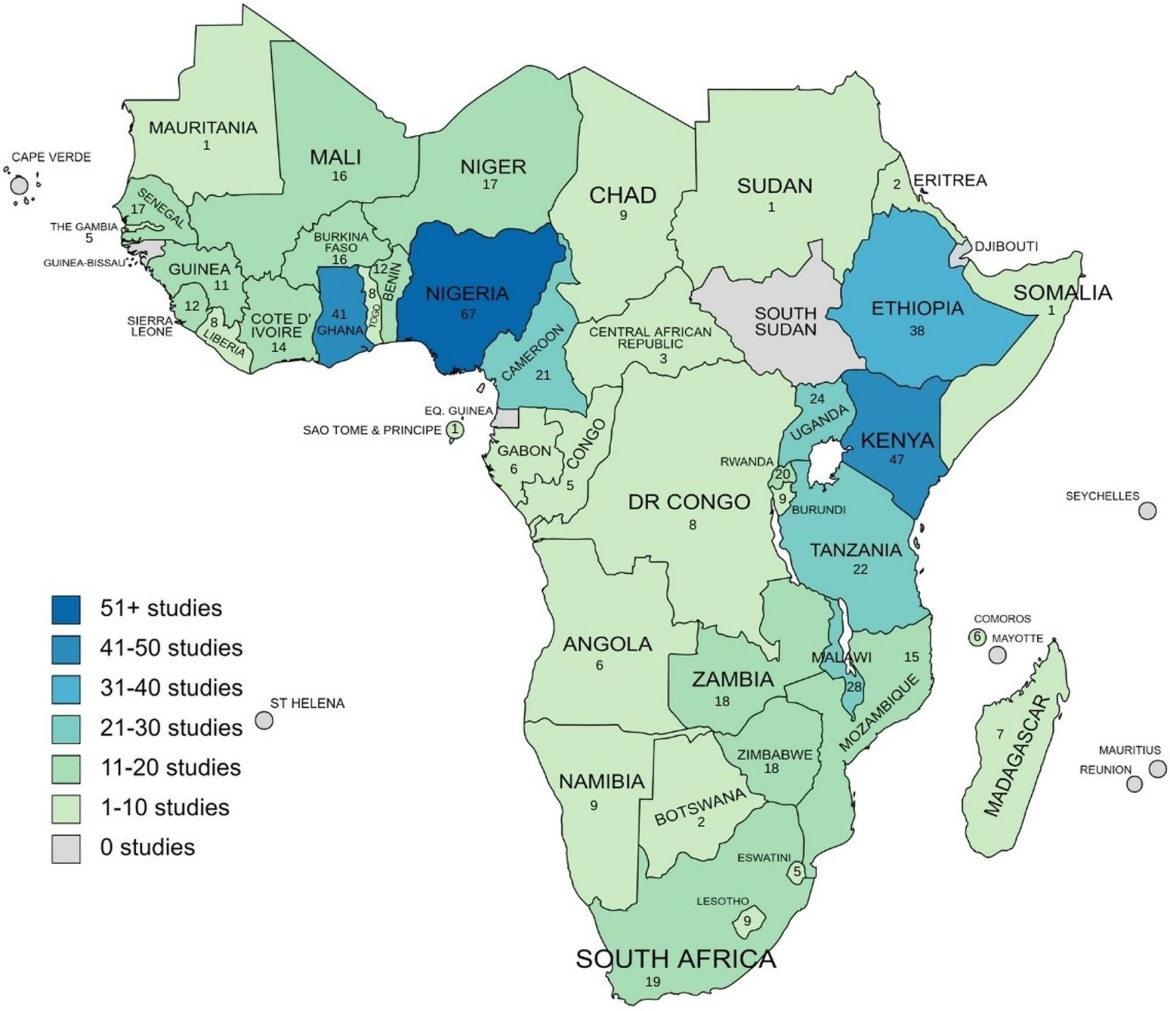
Included studies by country

In terms of geographic coverage of the 258 relevant pieces, most countries (91%) in SSA are represented, with West Africa having the largest concentration. Figure 2 shows the number of studies from each country.

Most pieces were published after the year 2000. Quantitative studies were overrepresented, constituting 154 of the 258 pieces. There were 64 qualitative studies and 36 mixed methods studies. Table 2 presents each determinant in terms of how many studies examined it and its role as a traditional/historical support or contemporary disrupter of high fertility. We have categorized the determinants from Table 2 into six overarching themes: economy and costs; marriage; mortality and health; the influence of others; education and status;  and demographic predictors. Within each of these six themes, we review the evidence on both traditional supports and contemporary disrupters of high fertility desires.

### Economy and costs

Children have long been viewed as economic resources for families and as providing security for parents in old age in SSA. Most studies (n = 63) found that viewing children as economic resources and children providing security for their parents in old age (n = 51) increased desired fertility. Having many children provides families with additional labor, and this economic value of children is especially high in agricultural societies, rural areas, and for poorer families. Amongst these groups, having many children can help with farming and trading. In the absence of governmental social support programs for the elderly in SSA, support and care from children remains important and not easily replaceable. Thus, having children to take care of elderly parents is important, and this is particularly true for women, especially if their spouse dies [24–27]. Having daughters is important for this reason, as most people expected that their daughters would be more likely to support them in old age than their sons [28]. One study found that having too many children can backfire, because one can only reap the benefits of old-age support if the children are raised properly. Thus, there may be quantity/quality tradeoffs where having too many children might render parents unable to invest enough effort into any one child and help secure their future success [29].

Increasing urbanization and changing economic conditions have altered the value of children. Almost all included studies on economy and costs (n = 60) found that recent economic changes, which have had the effect of increasing the cost of children, have decreased desired fertility. While children are still considered a source of wealth in more rural parts of SSA, they are increasingly seen as expensive burdens in urban areas where the cost of raising children and a higher overall cost of living outweighs potential economic returns. Widespread unemployment and increases in the cost of living have shaped the perceived value of children for urban residents of SSA. Among urban residents, education is perceived as improving future prospects for their children, a perception less prevalent in rural areas where farming and other agricultural occupations are still very common and require little, if any, formal schooling. Furthermore, many urban residents choose to have fewer children due to high schooling fees, so they can afford to invest more heavily in each child [30–33].

Several studies (n = 10) found that environmental factors—natural disasters, droughts, long-term climate change—have decreased desired fertility. Several mechanisms account for this. First is food shortages, leading to a recognition that fewer children can be supported [34–36]. Second, a decrease in cash crops and agricultural yield reduces the economic value of children [37–40]. Importantly, in many studies respondents indicated that while they continue to desire a large number of children, they had come to the conclusion that this was ill-advised under current conditions [15, 39, 41–43]. As a respondent in Agadjanian’s (2005) study states, “Now it seems that people don’t want to have many children, but it is not so. We want more children, but because of the [economic] situation we can’t” [15]. One can infer that improvements in economic conditions might lead to increased fertility rates.

Economic downturns often lead to increased migration for work and other opportunities. Migration is in turn associated with fertility desires, but this literature is limited (n = 4). Women married to successful migrants (e.g., those who found steady employment) were less likely to want to stop childbearing compared to women married to unsuccessful migrants [44–46]. Spousal migration may be important to consider in its influence on women’s fertility desires. In Rwanda, residents in high-migration areas were less likely to want a/another child than residents of low-migration areas, but this was likely driven by the proportion of refugees in high-migration areas [47].

### Marriage

Several pieces (n = 74) investigate fertility desires in relation to type of marital union (monogamous vs. polygynous). This collection of studies shows that polygyny both encourages higher fertility desires as well as serves as a mechanism for realizing high fertility desires. The bulk of studies (n = 47) found that polygyny was associated with higher fertility desires. Men can acquire additional wives with the purpose of achieving their desired family sizes and compositions. A small number (n = 6) found no significant difference in fertility desires between monogamous and polygynous unions. Additionally, preferences for male children may motivate entrance into polygynous unions, and high aggregate fertility desires have been found to be significantly associated with the presence and prevalence of polygyny in a community [90]. Qualitative studies provided more detailed insight into the structure and expectations of polygynous marriages and found that women expect and want to have many children because increased childbearing wins favor in competition between co-wives [50, 61, 62, 91]. The rank and placement of co-wives moderates individual women’s fertility desires with senior wives being more likely to desire no more children than junior ones, and this is shaped by age and parity [92]. In some cases, as they age and/or attain their desired fertility, senior wives may welcome a junior wife who can take on further childbearing responsibilities.

Apart from the matter of union type, several studies (n = 29) indicate that women's efforts to promote marital stability and secure spousal rights  were common reasons women wanted more children, with the caveat that too many children can also strain a marriage. A break-up of the union would place extra burden on the women for feeding, educating, and generally caring for their children. Some women felt pressure to conceive, especially after an abortion or fetal loss, to avoid marital strife [93]. When a wife does not want her husband to take another wife, she may increase her desired family size to match his [40]. Higher fertility can strengthen the wife’s relationship with her in-laws [94]. Women may also try to gain status within their marriage by having more children to persuade their new husband to accept children from a previous marriage [95].

Several studies (n = 15) found that spousal influence increases desired number of children, with both sexes willing to defer childbearing decisions to the more pronatalist partner. With men likely to be more pronatalist and with a wife’s fertility intentions more likely to be influenced by her husband’s fertility desires than vice versa, spousal influence often plays out in gendered ways [96]. Additionally, several studies (n = 17) found that spousal joint-decision making or discussion about family size decreased desired fertility for both spouses. In some cases, though, spousal discussion increased desired fertility when wives’ preferences were influenced by their spouse’s preference for more children [74, 97, 98]. Women who are older, have more than two children already, and desire to cease childbearing, have reported higher confidence in spousal communication on these issues, demonstrating that it is important to consider the potential implications of spousal discussion on fertility desires in the context of a highly patriarchal society [93].

Smaller ideal family sizes have been found among divorced/separated and widowed women compared to currently married ones [66], and remarriage often lends itself to higher fertility desires to accommodate the preferences of a new spouse [32]. Remarriage during and immediately after war—a survival strategy for women—can mean raising their desired fertility to have children with their new husband [95]. Specific circumstances of a marriage can also affect fertility desires. A higher age at marriage is associated with a lower desired number of children, and a larger age difference between spouses is associated with a lower desire to limit fertility compared to a smaller age difference [99].

### Mortality and health

Forty-four studies found that high rates of infant and child mortality increase desired fertility. Both men and women endorse having many children where mortality rates are high [100]. Those with direct experience of child death are more likely to engage in replacement and insurance strategies—having many children to ensure some survive to adulthood—in case future children also die [100, 101]. As SSA shifts from a high mortality to lower mortality regime, insurance strategies are no longer necessary in most contexts, but there can be cultural lag where structural conditions have changed but individual fertility desires and behaviors do not immediately adapt [102]. In the case of war or other traumatic violence, where mortality rates of children rise, included studies suggest that people may respond in one of two primary ways. They may wish to decrease childbearing or have no more children because of the effects of trauma on their physical and/or psychological well-being as well as a fear of bringing children into a world with greater uncertainty of future peace [47, 103–105]. Others may wish to have additional children to make up for ones that were killed, or for security in case of future war or violence which may subsequently increase mortality [95, 106]. The death of other family members also influences fertility desires. The death of a sibling is negatively associated with a preference for a large family [106], while the recent death of a parent led both men and women to desire more children [107]. When children’s parents die, other individuals may foster orphaned children. Fostering a child is associated with increased odds of reducing one’s ideal family size, indicating that foster children can contribute to achieving ideal family size in ways similar to biological children [108].

While we excluded studies that examined the fertility desires of HIV + individuals for reasons already described, we did include studies (n = 20) that examined how the context of the HIV/AIDS epidemic shapes fertility desires for HIV − individuals. There is a great deal of fear and stigma associated with HIV/AIDS, even for those who are not positive themselves. The sex of the participant influenced the effect of HIV/AIDS on desired fertility with women being more likely to fear HIV infection and, thus reduce desired fertility [101, 109]. Knowing someone with AIDS, high community mortality levels, and household child death were significant predictors of lower desired fertility for women but not for men [101].

Women’s perceptions of their own health also affect their fertility desires. Increased awareness of the detrimental health impacts of high parity and short birth spacing—awareness promoted by formal schooling and family planning programs—leads to reduced desired fertility [98, 110–114]. Women who participated in focus groups cited being tired of giving birth, too old, or of poor health as reasons for wanting to stop childbearing [115]. They also noted the toll that consecutive childbearing, breastfeeding and managing a large family takes on their bodies [93]. Difficult pregnancies and births may alter women’s initial fertility aspirations. When a woman thought that a pregnancy would threaten her health, her odds of wanting to stop childbearing increased [98]. Furthermore, women cited that the social value of their bodies declined with age and repeated deliveries; longer birth spacing and/or fewer births permitted them to remain attractive and physically fit [61].

### Influence of others

This theme encompasses the influence of other individuals or groups beside spouses on people’s fertility desires as well as the influence of social and cultural norms on fertility desires. Some studies found that larger families of origin were associated with a higher likelihood of desiring more children compared to respondents with smaller families of origin [44, 116]; this was particularly strong for men [116]. In one qualitative study on women who desired to be childfree, participants cited their large families of origin and amount of time spent mothering siblings and other children as a reason behind their decision [117].

Several studies (n = 22) found that desired fertility is influenced by family networks, and this effect varies according to which member of the family is the primary source of influence. For example, mothers-in-law or individuals’ own mothers may be particularly salient influences for increasing desired fertility [118]. Young people often felt the weight of their parents’ expectations that they would and should have children [119].

Many studies (n = 48) found that having many children can bring social status and prestige to families and communities. Large family size can symbolize and bring wealth, influence, and respect for men and women, and having many children can expand their social networks and may elevate them above their peers [48]. Often, rather than relying on their personal evaluation of resources, individuals rely on community perceptions and norms to determine their own fertility desires [120]. Observation of neighbors and other large families can influence individuals’ own ideal family sizes [120]. Some studies found that observing small families prompted participants to desire smaller families themselves and facilitated communication about family planning within their networks [15, 52, 67, 121, 122].

Three quantitative studies found no relationship between community influence and desired fertility; in these studies, community influence was operationalized as adolescent’s and women’s social interaction with their peers and/or community [96, 97, 123]. However, some individuals said their desired number of children was influenced by their local clinic(s), which might expand our conception of who is included in the ‘community’ and who might have influence on community members [120].

Another cultural and social norm that can influence fertility desires is gender/sex preference. In many communities, male offspring are more highly valued than female, and this preference has an effect on fertility desires, with extended childbearing in order to have male children a common practice [41]. Most studies (n = 32) found that persons who hold a sex preference have higher fertility desires. Five studies found that sex preference had little to no influence on desired fertility in settings where the desire for additional children was so comprehensive that children were highly valued and desired irrespective of sex [111, 124–127]. Even when a low overall number of children is desired, it is typically qualified by a preference for children of both sexes. Achieving this can produce desires for more children than otherwise wanted [65, 101, 118, 128].

Almost all articles that examined the influences of a lineage or clan (n = 27) found that it increased the desired number of children. For example, in Uganda, individuals belonging to families or ethnic groups with strong clan linkages wanted an average of 10.5 children, in contrast to those lacking such strong linkages who wanted 4.9 children [129]. This influence is particularly strong in patrilineal societies, where to maintain family lineage individuals must have at least one son [122, 130]. Two studies found that lineage/clan influence decreased the desired number of children, but these were cases of matrilineal societies where there is less concern about having many children and the desire to stop childbearing is more accepted [67, 131].

Findings from studies that examined the effect of ethnicity on fertility desires varied widely due to regional variation in included studies. Generally, ethnic groups within which large numbers of women preferred to have no more children are concentrated in East and Southern Africa [132, 133]. In West Africa, day-to-day life is more grounded in lineage relations, and this could be one reason desired fertility remains higher compared to other SSA regions [133]. A few studies that examined in-depth ethnic group differences in desired fertility focused on Nigeria and compared the Hausa/Fulani, Igbo/Ibo, Yoruba, and other ethnic groups, with the Igbo usually being the most likely to want more children [132, 134, 135]. For the Igbo, extended family is very important both socioculturally and economically and having large numbers of children helps to create a larger and more extensive network of family members [132].

Several studies (n = 55) found that the association between religion and desired fertility was influenced by specific religious affiliation and sex. In general, for both men and women, being Muslim is associated with higher fertility desires compared to other religious groups, although effects are sometimes stronger for men [63, 67, 74, 116, 136]. In qualitative studies, participants often talked about religion more broadly, in terms of God’s influence on childbearing. Children were seen as a gift from God that were not to be refused or otherwise influenced. Some participants felt that Muslims have large numbers of children because Allah wants them to and because their religion allows men to have more than one wife [41, 52, 113, 137]. Here, the intersection of polygyny and religion may create desires for large families. The influence of religion is stronger in rural areas [138].

Studies that explicitly measured the effects of colonialism, modernization and/or Western influence were uncommon. In some focus group discussions, participants mentioned that there was an increase in individualism and copying of Western behavior and thus traditional kin obligations may no longer be strong enough to sustain the demand for high fertility, especially as participants perceived that extended family support systems are eroding [139]. Small-family ideals were thought to be imported from other places and not ‘natural’ or ‘native’ to SSA [139].

### Education and status

A very common finding (n = 90) is a negative relationship between formal schooling and desired fertility. Two studies found that higher education was associated with high fertility [140, 141]. In these cases, the authors may be detecting short-term differentials: more schooling brings access to better jobs and increased income, producing a temporary increase in desired fertility, followed by a transitional period and a leveling out or decrease in the desire for more children. Some studies found that the relationship between education and desired fertility varied based on the sex of the participant, where the husband’s education had a larger effect on reducing fertility desires than the wife’s education [92, 142].

Higher education is a mechanism for women gaining status outside of the home and, more concretely, acquiring their own source of income, which in turn can affect their fertility desires. Findings regarding the effects of the status of women on fertility desires were somewhat disparate due to a lack of standardization in how the status of women was measured, including autonomy, empowerment, education, household profit contribution, and bargaining power. Several studies (n = 21) found that fertility desires fell with improved status. For example, women who contributed more than 50% to household expenditures are less likely to desire more than four children compared to women who contribute less than 50% [143]. On the other hand, when the status of women, particularly in rural and agricultural areas, is more directly tied to their ability to produce children, high fertility may be a way to achieve a higher status in the community [86, 118]. Qualitative work has found that women’s internal motivations for children include achieving happiness associated with motherhood and gaining respect and social status [80]. Only two studies found that placing a high value on children decreased desired fertility, and this was because participants felt that parents should put as many resources as they could toward fewer children [29, 122]. Put differently, because participants ascribed a high value to their children, they wanted to put as many resources toward them as they could which was increasingly possible with fewer numbers of children.

Increased education also increases knowledge of and access to family planning. Family planning and contraception use, or positive attitudes toward use, were associated with decreased desired fertility in 32 studies. Women who discussed family planning with their husbands were more likely to limit childbearing than those who never discussed it [106, 144]. Couples in which at least one partner was using contraception had lower demand for children compared with couples in which no partner was using contraception [145]. Additionally, knowledge of modern methods of contraception decreased desired number of children in some regions [145], but awareness of contraception on its own had no effect on desired number of children in others [113, 138, 146, 147]. This could be due to different methods of family planning promotion and/or different attitudes toward use. When family planning was operationalized at the level of clinic availability, access to these programs was associated with a decline in ideal family size [148]. Conversely, even in cases where contraceptive prevalence had increased, widespread resistance to modern contraception remains due to continued influence of traditional and cultural supports for high fertility and fears about modern contraception use [57].

Several studies (n = 8) found that general exposure to mass media decreased desired fertility. Watching TV and listening to the radio have been associated with decreased fertility desires and higher likelihood of wanting to stop childbearing [149, 150]. Many SSA countries have carried out mass media campaigns to specifically promote family planning and modern contraception. When people are exposed to mass media family planning messages specifically, this monotonically decreases the likelihood of desiring more children [151]. Some studies found that effects varied by media modality. For example, exposure to radio messages was associated with fewer desired children for both sexes, but watching TV was only associated with decreased desired fertility for women. This may be the result of gendered patterns in exposure to certain types of media [67, 87, 101, 152].

### Demographic predictors

A large number of included studies examined the relationship between age, parity, sex, and fertility desires. Age and parity are clearly related to each other (e.g., increasing age tends to increase parity), hence both determinants influence fertility desires in similar ways. For both of these predictors, the direction of the relationship depends on how fertility desires are operationalized. For example, in 39 studies, increasing age decreased desired number of children, with women aged 30 and above more likely than younger women to desire smaller numbers of children. On the other hand, increasing age increases ideal family size (n = 35) primarily due to post-hoc rationalization of existing children. Similarly, as parity increases (n = 47), desired fertility decreases, and the odds of wanting no more children increases with the number of living children. Women with higher numbers of living children are less likely to desire additional children compared to women with small numbers of children. As parity increases, ideal family size also increases (n = 14) due to post-hoc rationalization of past births as desired, regardless of whether they were desired at the time of the pregnancy. Seven studies found that age had no effect on desired number of children, but these studies either examined only adolescents (aged 15–19) or had small sample sizes.

Other findings related to age demonstrate that the association between age and fertility desires is more nuanced and can be influenced by current economic conditions and continued modernization. Young women in particular may be under more serious pressure than previous generations in light of harsh economic realities that would imply higher opportunity costs for their time and result in lower desired numbers of children [143].

There were consistent sex differences in desired family size (n = 43). Men were found to be more pronatalist and in favor of larger families compared to women, and this is in part due to the different roles that men and women tend to play within the home and in communities. Men often want large families because children can help with labor, while women often want smaller families because they are the primary caregivers and are more aware of the amount of work it takes to raise children [40].

Socioeconomic status (SES) and employment also affect fertility desires. Thirty-three studies indicated that as income and wealth increase, the desire to stop childbearing increases. Women in higher wealth quintiles are more likely to desire limiting childbearing than women in poorer wealth quintiles. A smaller number of studies found that SES both increased and decreased desired fertility when temporal increases in income or wealth were taken into account [42, 98, 99]. For example, as household income begins to increase, the demand for children rises because there is more financial security and an increased ability to support and care for higher numbers of children, but, after a higher threshold income level has been sustainably achieved, the desire for additional children usually dissipates [99].

Regarding employment, women who work and earn income tend to want fewer children than women who do not work. Most studies (n = 31) found that the relationship between employment and desired fertility was influenced by the type of employment, including whether it was characterized as formal versus informal or agricultural versus non-agricultural. Increases in the percentage of women working in formal employment reduced preferred family size, and women’s formal employment is consistently associated with decreased desired fertility [153]. Men’s employment, on the other hand, is less consistently associated with desired fertility in any single direction [154]. Some studies (n = 5) found no significant effect on desired fertility by type of employment, but these studies had very small numbers of women who worked outside of the home or earned income. Some studies found that married men who worked in agriculture were less likely to desire additional children than men who worked in non-agricultural jobs, but others found that women working in agriculture desired more children than their counterparts (non-agricultural) [65, 155].

## Discussion

Fertility desires in SSA have been intensely studied since the 1960s by social science and health scholars employing diverse methodologies and approaches. Collectively, this research literature has endeavored to understand how and why high fertility desires are generated and maintained in SSA. Our review of this literature shows that fertility desires are shaped by a myriad of factors, with results for any factor varying according to methodological approach and the country/population under examination. This produces some complexity, and even some contradictions, in the conclusions that can be drawn from the entire body of included studies. This feature notwithstanding, there are clear overarching patterns found in the supports and disrupters of high fertility desires. While contemporary changes in economic and family structures have influenced people’s desired fertility, causing them to shift their ideal family size downward, this is often viewed as a temporary adjustment to temporary conditions. The traditional and historical supports for high fertility, which have created strong ideological and cultural pronatalist values, have not disappeared entirely but, rather, have become more difficult to implement.

There is a great deal of evidence that increased autonomy of women, increased formal schooling of women, along with improved SES decreases fertility desires and positions women to more effectively implement their low[er] desires [112, 143, 156, 157]. The availability of family planning, and knowledge about modern methods and use, can also play a role in helping women implement lower fertility desires [106, 145, 148]. Most outside observers would evaluate these developments favorably. By contrast, other disrupters of high fertility desires are not evaluated positively by either outside observers or residents/communities in SSA. For example, poor economic prospects that make children difficult to afford and adequately care for, material deprivation, and scarcity of farmland are hardly positive developments.

We must acknowledge that improvements in the quality of life in SSA may result initially in increases in fertility, as improved conditions mean that individuals can afford and care for the additional children they continue to desire. There is evidence that such increases in desired fertility are temporary, and fertility desires (and fertility rates) decline as positive socioeconomic conditions continue and become normalized. This evidence is limited; how fertility desires and fertility rates in SSA respond to improvements in the quality of life is an important topic for continued research, especially using longitudinal designs.

We have identified several gaps in existing knowledge on fertility desires in SSA several of which can be attributed to methodological approaches. In our review, we find that 95% of included studies are cross-sectional. Longitudinal studies in the region are necessary to supplement existing cross-sectional data. We believe there should be increased, long-term funding that is necessary to undertake these kinds of projects. Additionally, it is often the case that quantitative or qualitative data alone provide a partial story. Quantitative studies predominate in our review, and many of these studies point to the need for their statistical findings to be supplemented with qualitative inquiry to better understand the mechanisms, contextualize relationships, and, ultimately, to understand *why.* Mixed methods approaches and increased in-depth qualitative inquiry to supplement existing quantitative survey data collection efforts will greatly contribute to knowledge production on fertility desires in the region and, importantly, provide residents of SSA the opportunity to tell their own stories and explain their lives directly. This is particularly relevant when trying to understand and/or measure uncertainty related to fertility desires, which has been difficult to achieve with existing quantitative data.

Another gap in existing research on fertility desires in SSA is the limited inclusion of men. In a patriarchal society where men are often seen as authoritative, knowledge of the ways in which ideologies and norms regarding masculinity impact women’s fertility desires and outcomes is particularly important. While the inclusion of men has grown over time, and many studies do include men or even focus on men alone, there is still much to be understood about the benefits of high fertility for men and their desires relative to their spouses. Studies show that spousal communication can be an important influence on fertility desires and family planning use, but how exactly spouses communicate with each other and how this influence works is not well understood [144, 158, 159]. Furthermore, existing survey measures could be refined to better identify and measure deliberate postponement and spacing of births that would provide more insight into the reproductive trajectories of women in SSA. The temporal dimension of fertility desires—how they evolve over time—is not well understood.

### Limitations

We note several limitations of this review. First, we did not specifically search for grey literature. However, we searched several databases that include reports, white papers, dissertations, and other types of publications beyond journal articles, as well as the personal collection of the principal investigator which includes several decades of research on fertility thus allowing us to capture some grey literature. Although we used a wide array of search terms to capture concepts of fertility and family size desires in SSA, some articles may not have been identified. Second, due to the limited language skills of the authors, we have only included studies published in English. This resulted in the exclusion of 46 citations published in French, the official language in several SSA countries. This could have led to the exclusion of work from authors native to the region and could explain why several countries with high fertility rates (e.g. Niger, Democratic Republic of Congo) are represented by few studies. However, almost half of the 46 excluded citations were either DHS reports or duplicates of items included in English and would have therefore been excluded for other reasons. Third, due to the COVID-19 pandemic and resulting library closures and lending restrictions, we were unable to obtain the full texts of several studies which may have been relevant for this review.

## Conclusion

Based on our review of 258 articles, we conclude that traditional and historical supports for high fertility continue to influence fertility desires in SSA up to the present. Concurrently, there are important contemporary disrupters of those supports that are depressing fertility desires in the region. These disrupters could be viewed as positive (e.g., increased education and autonomy for women) and negative (e.g., poor economy and job prospects) in nature. A striking conclusion of this review is that declines in desired fertility are  often a response to poor structural conditions; it follows that when those conditions improve, fertility desires and rates may respond positively, because high fertility remains highly valued in many contexts. Given the limitations of existing research and calls for methodological diversity, we believe that increased qualitative inquiry to supplement existing survey research and more longitudinal research in this area is needed. We call for future research that  centers the experiences and desires of SSA men and women without implying Western ideals or understandings of fertility and its benefits and consequences.

## Supplementary Information

**Additional file 1: Appendix A Table S1**. Preferred Reporting Items for Systematic reviews and Meta-Analyses extension for Scoping Reviews (PRISMA-ScR) Checklist.** Appendix B Table S2**. Sample Search Strategy in PubMed.** Appendix C Table S3**. Overview of variables’ relationship to fertility desires found in studies.

## Data Availability

The extracted data used for this scoping review are available from the corresponding author on reasonable request.

## Abbreviation

SSA: Sub-Saharan Africa
