# Supplementary material for: Traditional supports and contemporary disrupters of high fertility desires in sub-Saharan Africa: a scoping review
Source: Reprod Health. 2023 Jun 6;20:86. doi: 10.1186/s12978-023-01627-7 (PMC10242605; doi:10.1186/s12978-023-01627-7)
Supplement: Supplementary file 1 — Additional file 1: Appendix A Table S1. Preferred Reporting Items for Systematic reviews and Meta-Analyses extension for Scoping Reviews (PRISMA-ScR) Checklist. Appendix B Table S2. Sample Search Strategy in PubMed. Appendix C Table S3. Overview of variables’ relationship to fertility desires found in studies. [file 12978_2023_1627_MOESM1_ESM.docx]

**APPENDIX A**

Table S1. Preferred Reporting Items for Systematic reviews and Meta-Analyses extension for Scoping Reviews (PRISMA-ScR) Checklist

| **SECTION** | **ITEM** | **PRISMA-ScR CHECKLIST ITEM** | **REPORTED ON PAGE #** |
| --- | --- | --- | --- |
| **TITLE** | | | |
| Title | 1 | Identify the report as a scoping review. | 1 |
| **ABSTRACT** | | | |
| Structured summary | 2 | Provide a structured summary that includes (as applicable): background, objectives, eligibility criteria, sources of evidence, charting methods, results, and conclusions that relate to the review questions and objectives. | 1 |
| **INTRODUCTION** | | | |
| Rationale | 3 | Describe the rationale for the review in the context of what is already known. Explain why the review questions/objectives lend themselves to a scoping review approach. | 2 |
| Objectives | 4 | Provide an explicit statement of the questions and objectives being addressed with reference to their key elements (e.g., population or participants, concepts, and context) or other relevant key elements used to conceptualize the review questions and/or objectives. | 2 |
| **METHODS** | | | |
| Protocol and registration | 5 | Indicate whether a review protocol exists; state if and where it can be accessed (e.g., a Web address); and if available, provide registration information, including the registration number. | 3 |
| Eligibility criteria | 6 | Specify characteristics of the sources of evidence used as eligibility criteria (e.g., years considered, language, and publication status), and provide a rationale. | 3 |
| Information sources* | 7 | Describe all information sources in the search (e.g., databases with dates of coverage and contact with authors to identify additional sources), as well as the date the most recent search was executed. | 3 |
| Search | 8 | Present the full electronic search strategy for at least 1 database, including any limits used, such that it could be repeated. | Appendix B |
| Selection of sources of evidence† | 9 | State the process for selecting sources of evidence (i.e., screening and eligibility) included in the scoping review. | 3 |
| Data charting process‡ | 10 | Describe the methods of charting data from the included sources of evidence (e.g., calibrated forms or forms that have been tested by the team before their use, and whether data charting was done independently or in duplicate) and any processes for obtaining and confirming data from investigators. | 4 |
| Data items | 11 | List and define all variables for which data were sought and any assumptions and simplifications made. | 3-4 |
| Critical appraisal of individual sources of evidence§ | 12 | If done, provide a rationale for conducting a critical appraisal of included sources of evidence; describe the methods used and how this information was used in any data synthesis (if appropriate). | N/A |
| Synthesis of results | 13 | Describe the methods of handling and summarizing the data that were charted. | 4 |
| **RESULTS** | | | |
| Selection of sources of evidence | 14 | Give numbers of sources of evidence screened, assessed for eligibility, and included in the review, with reasons for exclusions at each stage, ideally using a flow diagram. | 5 |
| Characteristics of sources of evidence | 15 | For each source of evidence, present characteristics for which data were charted and provide the citations. | Appendix C |
| Critical appraisal within sources of evidence | 16 | If done, present data on critical appraisal of included sources of evidence (see item 12). | N/A |
| Results of individual sources of evidence | 17 | For each included source of evidence, present the relevant data that were charted that relate to the review questions and objectives. | Appendix C |
| Synthesis of results | 18 | Summarize and/or present the charting results as they relate to the review questions and objectives. | 4-11 |
| **DISCUSSION** | | | |
| Summary of evidence | 19 | Summarize the main results (including an overview of concepts, themes, and types of evidence available), link to the review questions and objectives, and consider the relevance to key groups. | 11-12 |
| Limitations | 20 | Discuss the limitations of the scoping review process. | 12 |
| Conclusions | 21 | Provide a general interpretation of the results with respect to the review questions and objectives, as well as potential implications and/or next steps. | 12 |
| **FUNDING** | | | |
| Funding | 22 | Describe sources of funding for the included sources of evidence, as well as sources of funding for the scoping review. Describe the role of the funders of the scoping review. | 12 |

JBI = Joanna Briggs Institute; PRISMA-ScR = Preferred Reporting Items for Systematic reviews and Meta-Analyses extension for Scoping Reviews.

* Where *sources of evidence* (see second footnote) are compiled from, such as bibliographic databases, social media platforms, and Web sites.

† A more inclusive/heterogeneous term used to account for the different types of evidence or data sources (e.g., quantitative and/or qualitative research, expert opinion, and policy documents) that may be eligible in a scoping review as opposed to only studies. This is not to be confused with *information sources* (see first footnote).

‡ The frameworks by Arksey and O’Malley (6) and Levac and colleagues (7) and the JBI guidance (4, 5) refer to the process of data extraction in a scoping review as data charting*.*

§ The process of systematically examining research evidence to assess its validity, results, and relevance before using it to inform a decision. This term is used for items 12 and 19 instead of "risk of bias" (which is more applicable to systematic reviews of interventions) to include and acknowledge the various sources of evidence that may be used in a scoping review (e.g., quantitative and/or qualitative research, expert opinion, and policy document).

**APPENDIX B**

Table S2. Sample Search Strategy in PubMed.

| **Population^[[1]](#endnote-1)^, Concept, Context (PCC)** | **Search** | **Search terms** | **Number of results** |
| --- | --- | --- | --- |
| **Concept:** Fertility desires | #1 | "Fertility"[Mesh:NoExp] OR fertility[tiab] OR "family size"[tiab] OR parity[mesh] OR parity[tiab] OR parenthood[tiab] OR childbearing[tiab] OR "Family Characteristics"[Mesh:NoExp] | 197,755 |
|  | #2 | Prefer*[tiab] OR desire*[tiab] OR Motivation[mesh] OR motiv*[tiab] OR ideal[tiab] OR incentive*[tiab] OR determinant*[tiab] OR intent*[tiab] OR intend*[tiab] OR goal*[tiab] OR behavio*[tiab] OR "decision making"[mesh] OR "decision making"[tiab] | 3,086,422 |
|  | #3 | #1 AND #2 | 40,626 |
| **Context:**  Sub-Saharan Africa | #4 | "Africa South of the Sahara"[mesh] OR "sub-Saharan Africa"[tiab] OR Angola[tiab] OR Benin[tiab] OR Botswana[tiab] OR "Burkina Faso"[tiab] OR Burundi[tiab] OR Cameroon[tiab] OR "Cape Verde"[tiab] OR "Cabo Verde"[tiab] OR "Central African Republic"[tiab] OR Chad[tiab] OR Comoros[tiab] OR Congo[tiab] OR "Congo Republic"[tiab] OR "Republic of Congo"[tiab] OR DRC[tiab] OR "Democratic Republic of Congo"[tiab] OR "Côte d'Ivoire"[tiab] OR "ivory coast"[tiab] OR Djibouti[tiab] OR "Equatorial Guinea"[tiab] OR Eritrea[tiab] OR Ethiopia[tiab] OR Gabon[tiab] OR Gambia[tiab] OR Ghana[tiab] OR Guinea[tiab] OR "Guinea-Bissau"[tiab] OR Kenya[tiab] OR Lesotho[tiab] OR Liberia[tiab] OR Madagascar[tiab] OR Malawi[tiab] OR Mali[tiab] OR Mauritania[tiab] OR Mauritius[tiab] OR Mozambique[tiab] OR Namibia[tiab] OR Niger[tiab] OR Nigeria[tiab] OR Réunion[tiab] OR Rwanda[tiab] OR ("Sao Tome"[TIAB] AND Principe[Title/Abstract]) OR Senegal[tiab] OR Seychelles[tiab] OR "Sierra Leone"[tiab] OR Somalia[tiab] OR "South Africa"[tiab] OR Sudan[tiab] OR "SOUTH SUDAN"[tiab] OR Swaziland[tiab] OR eSwatini[tiab] OR Tanzania[tiab] OR Togo[tiab] OR Uganda[tiab] OR "Western Sahara"[tiab] OR Zambia[tiab] OR Zimbabwe[tiab] OR Zaire[tiab] OR "South West Africa"[tiab] | 422,452 |
|  | #5 | #3 AND #4 | 4,222 |
|  | #6 | ("1990/01/01"[PDAT] : "2020/12/31"[PDAT]) | 1,328,666 |
|  | #7 | #5 AND #6 | 256 |

**APPENDIX C**

Table S3. Overview of variables’ relationship to fertility desires found in the studies.

| **Traditional Supports** |  |  |  |
| --- | --- | --- | --- |
| **Variable** | **Positive association with fertility desires** | **Negative association with fertility desires** | **No association with fertility desires** |
| Age | Althaus, 1997; Ambel, 2007; Angawa, 1997; Bankole et al., 2004; Baylies C., 2000; Bekele, 2004; Benefo, 1990; CampbellE K, 1993; Capo-Chichi, 1999; Ettyang, 1997; Ezra, 1997, 2001; Heinz & Roth, 2019; Hollos M & Larsen U, 2004; Isiugo-Abanihe, 1994a; Kebede et al., 2021; Kiriti & Tisdell, 2005; LeVine & LeVine, 1998; Mahmud, 2017; Muhoza, 2019; Nwogu, 2006; O. C. Odimegwu, 1994; Odusola & and others, 1998; Okafor et al., 2021; Owoo et al., 2015; Owoo & Lambon-Quayefio, 2021; Rutayisire et al., 2013; Sam et al., 2005; Shapiro, 2018; Solanke, 2017; Suda, 1997; F. Tadesse & Headey, 2010; Takyi, 1993; Upadhyay & Karasek, 2012; Yeatman et al., 2013 | Agadjanian, 1995; Ahinkorah et al., 2020, 2021;Amin et al., 1992; Anonymous, 2016; Avogo, 2008; Banda, 1990; Berhanu, 1994; Dibaba, 2009; Ekouevi, 1992; Ewemooje et al., 2020; Fosu et al., 2013; Gasafari, 2006; Green, 1997; Hayford & Agadjanian, 2012; Isiugo-abanihe, 1997; Kodzi, 2009; Kodzi et al., 2010, 2012; Lemessa & Wencheko, 2014; Levin, 2001; Mahy, 2000; Mashara, 2016; Matovu J.K.B. et al., 2017; McGinn, 2004; Modile, 2017; Musalia, 2000; Muvandi, 2003; Novignon J. et al., Nwakeze, 2007; Obiero, 1998; Oladosu, 1999; Omariba, 2006; Oyediran, 2006; Oyediran & Isiugo-Abanihe, 2002; Snow et al., 2013; Togunde & Newman, 2005; Uchudi, 1999; Van de Walle F. & Maiga M., 1991; Westoff & Bietsch, 2015 | Adler et al., 2017; Akinyoade, 1999; Herman et al., 2011; Ibrahim & Arulogun, 2020; Perkins, 1997; Poukouta & Fapohunda, 1997; Saila-Ngita et al., 2003 |
| Religion | Abdi et al., 2021; Adewole et al., 2020; Adongo et al., 1997; Akeju et al., 2021; Akinyoade, 1999; Ampofo, 2002; Avogo, 2008; Beyeza-Kashesya et al., 2010; Caplan, 1995; Dahl-Jorgensen, 1990; Heinz & Roth, 2019; Hollos M & Larsen U, 2004; Hough, 2008; L. D. G. Ibisomi, 2008; L. Ibisomi & Odimegwu, 2011; Kahsay et al., 2018; Lemessa & Wencheko, 2014; LeVine & LeVine, 1998; Lloyd, 1993; Modile, 2017; Muhoza, 2019; Mukasa, 2015; E. Nwogu, 2006; Oyediran, 2006; Price, 1995a, 1995b; Randall & LeGrand, 2003; Sahleyesus et al., 2009; Samandari et al., 2019; Sinai et al., 2019; Snow et al., 2013; Telake, 2006; Van de Walle F. & Maiga M., 1991; Wilson, 1998 | Bougma et al., 2021; Short & Kiros, 2002 | Agadjanian, 1998; Ibrahim, 2020c; Kalesanwo & Adenuga Emmanuel, 2009; Kirumira, 1996; Olaogun A et al., 2009 |
| Place of residence | Adebimpe et al., 2011; Adebowale & Palamuleni, 2015; Ahinkorah et al., 2020, 2021; Althaus, 1997; Angawa, 1997; Baylies C., 2000; Capurchande et al., 2017; Du Plessis, 1996; Ettyang, 1997; Kebede et al., 2021; Mahmud, 2017; Mulenga et al., 2018; E. Nwogu, 2006; Odimegwu, 1994; Odusina et al., 2020; Odusola & and others, 1998; Olenick, 1998; Oyediran & Isiugo-Abanihe, 2002; Perkins, 1997; Rutayisire et al., 2013; I. Speizer, 1995; Staveteig, 2011; Takyi, 1993; Woldemicael, 2009 | Acsadi & Johnson-Acsadi, 1990; Agadjanian, 1998; Anonymous, 2016; Babalola et al., 2018; Banda, 1990; Benefo, 1990; Campbell & Campbell, 1997; Capo-Chichi, 1999; Capo-chichi & Juarez, 2001; Derose & Ezeh, 2005; Ekouevi, 1992; Ewemooje et al., 2020; Ezra, 1997; Fosu et al., 2013; Gasafari, 2006; Grieser et al., 2001; Heinz & Roth, 2019; Imo et al., 2014; Isiugo-abanihe, 1997; Mashara, 2016; McGinn, 2004; Nwakeze, 2007; Oladosu, 1999; Omariba, 2006; Owoo et al., 2015; Poukouta & Fapohunda, 1997; Randall & LeGrand, 2003; Shapiro, 2018; Short & Kiros, 2002; Snow et al., 2013; Westoff & Bietsch, 2015 | Agadjanian, 1995; Hollos & Larsen, 1992; Isiugo-Abanihe, 1994b; Kahsay et al., 2018; Modile, 2017; Muvandi, 2003; Novignon J. et al., 2019 |
| Polygyny | Adewole et al., 2020; Ampofo, 2002; Angawa, 1997; Anonymous, 2016; Avogo, 2008; Baschieri et al., 2013;Bawah et al., 1999; Benefo, 1990;Bledsoe, 1990; Bolten & Marcantonio, 2021; Calvès & Meekers, 1999; Caplan, 1995; Capo-Chichi, 1999; DeRose et al., 2002; DeRose, 2007; Dim, 2018; Dodoo, 1998; Dow Jr. et al., 1997; Dybdahl & Hundeide, 1998; Ettyang, 1997; Eyayou et al., 2004; Ezeh, 1997; Fapohunda & Todaro, 1988; Green, 1997; Hollos & Larsen, 1992; Hollos M & Larsen U, 2004; Isiugo-Abanihe, 1994b; C. Izugbara et al., 2010;C. O. Izugbara & Ezeh, 2010; Kahsay et al., 2018; Karp C. et al., 2020; Madhavan, 1998; Mashara, 2016;Milazzo, 2014; Muvandi, 2003; Ndiaye et al., 2018; Nwogu, 2006; Nwokocha, 2007; Odusina et al., 2020; Odusola & and others, 1998; Olalekan, 2013; Oyediran, 2006; Perkins, 1997; Price, 1995a, 1995b; Randall & LeGrand, 2003; Ratcliffe et al., 2000; Sinai et al., 2019;I. Speizer, 1999; I. S. Speizer, 1995; Teye, 2013; Ukaegbu, 2013 | Agadjanian, 1995, 1998; Hayford & Agadjanian, 2012; Isiugo-abanihe, 1997 | Ettyang, 1997; Novignon J. et al., 2019; Oyediran & Isiugo-Abanihe, 2002; Poukouta & Fapohunda, 1997; (Saila-Ngita et al., 2003); Umoh et al., 2012 |
| Parity | Angawa, 1997; Bachan, 2015; Benefo, 1990; Cottrell, 2010; Isiugo-abanihe, 1997;Mahmud, 2017; Modile, 2017; Muhoza, 2019; Obembe et al., 2018; Owoo et al., 2015; Saila-Ngita et al., 2003; Solanke, 2017; Togunde & Newman, 2005; Upadhyay & Karasek, 2012; Westoff & Bietsch, 2015; Yeatman & Sennott, 2014 | Acsadi & Johnson-Acsadi, 1990; Adler et al., 2017; Agadjanian, 1995, 1998; Ahinkorah et al., 2020, 2021; Akeju et al., 2021; Amin et al., 1992; Anonymous, 2016; Avogo, 2008; Babalola et al., 2018; Banda, 1990; Berhanu, 1994; Bougma et al., 2021; Casterline & Agyei-Mensah, 2017; Derose & Ezeh, 2005; Dybdahl & Hundeide, 1998; Ekouevi, 1992; Ettyang, 1997; Ewemooje et al., 2020; Fayehun et al., 2020; Fosu et al., 2013; Green, 1997; Hayford & Agadjanian, 2012, 2012, 2017, 2019; Kodzi, 2009;Kodzi et al., 2010, 2012; Levin, 2001; Mahy, 2000; Mashara, 2016; Matovu J.K.B. et al., 2017; McGinn, 2004; Musalia, 2000; Muvandi, 2003; Obiero, 1998; Omariba, 2006; Oyediran & Isiugo-Abanihe, 2002; Sathiya Susuman A. et al., 2014; I. S. Speizer, 1995; Suda, 1997; Takyi, 1993; Thiede et al., 2020; Uchudi, 1999; Woldemicael, 2009 |  |
| Child/Infant mortality | Adongo et al., 1997; Akinyoade, 1999; Angawa, 1997; Bauer et al., 2006; Bawah et al., 1999; Benefo, 1990; Bhargava A., 2007;CampbellE K, 1993; Caplan, 1995; Capo-Chichi, 1999; Dibaba, 2009; Ekouevi, 1992; Ettyang, 1997; Eyayou et al., 2004; Ezra, 1997;Farmer et al., 2015; Fitaw et al., 2004; Kidman & Anglewicz, 2014;L. D. G. Ibisomi, 2008; C. Izugbara et al., 2010; A. M. Jensen, 1991; Kahsay et al., 2018; Kahansim et al., 2013;Kodzi et al., 2012; LeGrand et al., 2003; Lemessa & Wencheko, 2014; LeVine & LeVine, 1998; Levin, 2001; Lloyd, 1993; Lowe et al., 2021; Mahy, 2000;Mairiga et al., 2010; Moyo & Mbizvo, 2004; Muhoza, 2019; Muvandi, 2003;Ndiaye et al., 2018; Novignon J. et al., 2019; Nwogu, 2006; O. C. Odimegwu, 1994; Owoo et al., 2015; Perkins, 1997; Randall & LeGrand, 2003; Rutayisire et al., 2013; Smith, 2004; Staveteig, 2011; Suda, 1997; B. Tadesse & Asefa, 2002; Telake, 2006; Westoff & Bietsch, 2015, 2015; Wilson, 1998; Woldesenbet, 2010; Yeatman et al., 2013 | Adewole et al., 2020; Bachan, 2015; Campbell & Campbell, 1997; Capo-chichi & Juarez, 2001; Rutayisire et al., 2013; Shapiro, 2018; Shapiro & Tenikue, 2017 | Agadjanian, 1995; Poukouta & Fapohunda, 1997; Sathiya Susuman A. et al., 2014 |
| Sex preference | Adewole et al., 2020; Ambel, 2007; Angawa, 1997; Anonymous, 2016; Ayoub, 2003; Bawah et al., 1999; Beyeza-Kashesya et al., 2010; CampbellE K, 1993; Caplan, 1995; Capo-Chichi, 1999; Dibaba, 2009; Ettyang, 1997; Fayehun et al., 2020; Gasafari, 2006; Isiugo-Abanihe, 1994b; A.-M. Jensen, 2015; Kahsay et al., 2018; Kirumira, 1996; LeVine & LeVine, 1998; Mahy, 2000;Milazzo, 2014; Muvandi, 2003; Nwokocha, 2007; Obembe et al., 2018;OGBONNA & MAMMAN, 1990; Okafor et al., 2021; Olaogun A et al., 2009; Oshodin & Ujiro, 2007; Price, 1995b; Robilliard, 2020;Short & Kiros, 2002;Smith, 1999, 2004; Teye, 2013; Togunde & Newman, 2005 |  | Acsadi & Johnson-Acsadi, 1990; Imo et al., 2014; Kalesanwo & Adenuga Emmanuel, 2009; Mairiga et al., 2010; Trinitapoli & Yeatman, 2018 |
| Children as economic resource | Adewole et al., 2020; Adongo et al., 1997; Banda, 1990; Barden-O’Fallon, 2005; Bekele, 2004; Beyeza-Kashesya et al., 2010;Bolten & Marcantonio, 2021; Calvès & Meekers, 1999; Caplan, 1995; Capo-Chichi, 1999; Dahl-Jorgensen, 1990; Dybdahl & Hundeide, 1998; Ekouevi, 1992; Fairlamb, 1990; Fairlamb & Nieuwoudt, 1991; Fitaw et al., 2004; Garver, 2017; Gasafari, 2006; Heinz & Roth, 2019; Hollos & Larsen, 1992; Hough, 2008; Hussain, 1991; Isiugo-Abanihe, 1994b; C. O. Izugbara & Ezeh, 2010; A. M. Jensen, 1991; Joekes, 1994; Kahansim et al., 2013; Kahsay et al., 2018; Kiriti & Tisdell, 2005; Kirumira, 1996; Kurth et al., 2015; LeGrand et al., 2003; Lloyd, 1993; Lowe et al., 2021; Mahy, 2000; Mayer B & Trommsdorff G, 2010; Telake, 2006;Ndiaye et al., 2018; Norris et al., 2019; Okafor et al., 2021; Oshodin & Ujiro, 2007; Randall & LeGrand, 2003; Sahleyesus et al., 2009; Sam et al., 2005; Sathiya Susuman A. et al., 2014; Smith, 1999, 2004; Spjeldnaes et al., 2007; Suda, 1997; B. Tadesse & Asefa, 2002; Teye, 2013; Togunde & Newman, 2005; Vohra, 2014; Wilson, 1998 |  | Bauer et al., 2006 |
| Security in old age | Adewole et al., 2020; Adongo et al., 1997; Ambel, 2007; Ampofo et al., 2009; Banda, 1990; Barden-O’Fallon, 2005; Beyeza-Kashesya et al., 2010; Calvès & Meekers, 1999; Caplan, 1995; Capo-Chichi, 1999; Capurchande et al., 2017; Dybdahl & Hundeide, 1998; Fairlamb, 1990; Fairlamb & Nieuwoudt, 1991; Fitaw et al., 2004; Garver, 2017, 2018; Green, 1997; Grieser et al., 2001; Heinz & Roth, 2019; Hough, 2008; L. D. G. Ibisomi, 2008; Isiugo-Abanihe, 1994b; C. O. Izugbara & Ezeh, 2010; Kahansim et al., 2013; Kahsay et al., 2018; Kiriti & Tisdell, 2005; Kurth et al., 2015; LeVine & LeVine, 1998; Lloyd, 1993; Mahy, 2000; Mairiga et al., 2010; Ndiaye et al., 2018; Norris et al., 2019; Nwokocha, 2007; Oshodin & Ujiro, 2007; Owoo et al., 2015; Price, 1995a; Randall & LeGrand, 2003; Sahleyesus et al., 2009; Sam et al., 2005; Smith, 2004; Spjeldnaes et al., 2007; Suda, 1997; B. Tadesse & Asefa, 2002; Telake, 2006; Teye, 2013; Togunde & Newman, 2005; Vohra, 2014; Wilson, 1998; Woldesenbet, 2010 | Ibrahim, 2020b |  |
| Value of children | Adewole et al., 2020; Adongo et al., 1997; Ampofo et al., 2009; Angawa, 1997; Barden-O’Fallon, 2005; Bawah et al., 1999; Calvès & Meekers, 1999; Caplan, 1995; Capo-Chichi, 1999; Dybdahl & Hundeide, 1998; Dyer et al., 2008; Eyayou et al., 2004; Fitaw et al., 2004; Grieser et al., 2001; Heinz & Roth, 2019; Hollos & Larsen, 1992, 1997; Hough, 2008; L. D. G. Ibisomi, 2008; Isiugo-Abanihe, 1994a; C. O. Izugbara & Ezeh, 2010; A. M. Jensen, 1991; Kahsay et al., 2018; Kannae & Pendleton, 1994; Karp C. et al., 2020; LeVine & LeVine, 1998; Lloyd, 1993; Mairiga et al., 2010; Mathur et al., 2016; Mayer B & Trommsdorff G, 2010; Mukasa, 2015; Norris et al., 2019; Nwokocha, 2007; Oshodin & Ujiro, 2007; Pierotti, 2013; Price, 1995a; Randall & LeGrand, 2003; Ratcliffe et al., 2000; Sahleyesus et al., 2009; Sam et al., 2005; Smith, 1999, 2004; Spjeldnaes et al., 2007; Suda, 1997; Swartz et al., 2018; Telake, 2006; Teye, 2013; Togunde & Newman, 2005; Vohra, 2014 | Ibrahim, 2020c; Towriss, 2014 |  |
| Community influence | Ampofo, 2002; Ampofo et al., 2009; Angawa, 1997; Barden-O’Fallon, 2005; Capo-Chichi, 1999; Capurchande et al., 2017; DeRose et al., 2002a; Garver, 2017; Geleta, 2018; Green, 1997; Grieser et al., 2001; Ibrahim, 2020c; C. O. Izugbara & Ezeh, 2010; A. M. Jensen, 1991; A.-M. Jensen, 2015, 2017; Karp C. et al., 2020; Kirumira, 1996; LeGrand et al., 2003; Levin, 2001; LeVine & LeVine, 1998; Mahy, 2000; Mairiga et al., 2010; Mathur et al., 2016; Mukasa, 2015; Ndiaye et al., 2018; Norris et al., 2019; J. N. Nwogu et al., 2021; Nwokocha, 2007; Oladosu, 1999; Owoo et al., 2015; Pierotti, 2013; Sahleyesus et al., 2009; Sam et al., 2005; Samandari et al., 2019; Smith, 1999, 2004; Swartz et al., 2018; Telake, 2006; Teye, 2013; Vohra, 2014 | Agadjanian, 2005; Avogo, 2008; Caplan, 1995; Musalia, 2000; Towriss, 2014 | Herman et al., 2011; Kahansim et al., 2013; Kodzi, 2009 |
| Marital stability | Agadjanian, 1998, 2005; Ambel, 2007; Ampofo, 2002; Banda, 1990; Baynes et al., 2020; Bolten & Marcantonio, 2021; Caplan, 1995; Capo-Chichi, 1999; Cottrell, 2010; Dybdahl & Hundeide, 1998; Grieser et al., 2001; Harrison & Montgomery, 2001; Hayford & Agadjanian, 2017; Hough, 2008; C. Izugbara et al., 2010, 2010; Kirumira, 1996; Lloyd, 1993; Mahy, 2000; Norris et al., 2019; Nwogu, 2006; Nwokocha, 2007; Ratcliffe et al., 2000; Sahleyesus et al., 2009; Sam et al., 2005; Staveteig, 2011; Swartz et al., 2018;Ukaegbu, 2013; Vohra, 2014; Woldesenbet, 2010 | Adebowale & Palamuleni, 2015; Hollos M & Larsen U, 2004; Isiugo-Abanihe, 1994b; Levin, 2001; Towriss, 2014; Towriss et al., 2020 |  |
| Sex of participant | Banda, 1990; Bankole et al., 2004; Bankole & Singh, 1998; Benefo & Pillai, 2005; Bolten & Marcantonio, 2021;Geleta, 2018; Heinz & Roth, 2019; Ibrahim, 2020; Isiugo-Abanihe, 1994a; Matovu J.K.B. et al., 2017;Mesfin, 2002;Odusola & and others, 1998; (Olenick, 1998) Ratcliffe et al., 2000; Reynar, 2000; Ukaegbu, 2013 | Agadjanian, 2005; Bauer et al., 2006; Derose & Ezeh, 2005; Green, 1997; Herman et al., 2011; Telake, 2006 | Campbell & Campbell, 1997; Capo-chichi & Juarez, 2001; Kahansim et al., 2013; Kirumira, 1996; Kouame & Schellekens, 2002 |
| Lineage/clan influence | Abdi et al., 2021; Adongo et al., 1997; Angawa, 1997; Banda, 1990; Bauer et al., 2006; Capo-Chichi, 1999; Ekouevi, 1992; Grieser et al., 2001; Hough, 2008; L. D. G. Ibisomi, 2008; Isiugo-Abanihe, 1994a; Kahsay et al., 2018; LeGrand et al., 2003; LeVine & LeVine, 1998; Nwokocha, 2007; Okafor et al., 2021; Olaogun A et al., 2009; Price, 1995b; Sahleyesus et al., 2009; Sam et al., 2005; Smith, 1999, 2004; Spjeldnaes et al., 2007; Suda, 1997; Telake, 2006; Togunde & Newman, 2005; Towriss, 2014 | Avogo, 2008; Elleamoh & Dake, 2019 |  |
| Family influence | Agadjanian, 1995; Barden-O’Fallon, 2005; Beyeza-Kashesya et al., 2010; Bimha & Chadwick, 2016; Burgess et al., 2020; Ibrahim, 2020c; A.-M. Jensen, 2015; Karp C. et al., 2020; Kirumira, 1996; LeGrand et al., 2003; Levin, 2001; Lloyd, 1993; Madhavan, 1998; Madhavan & Bledsoe, 2001; Mahy, 2000; Modile, 2017; Mosha et al., 2013; Moultrie et al., 2001; Nwokocha, 2007; Sam et al., 2005; Samandari et al., 2019; Shreffler & Dodoo, 2009; Smith, 1999, 2004; Spjeldnaes et al., 2007; Telake, 2006; Teye, 2013; Towriss, 2014 | Avogo, 2008 | Oladosu, 1999 |
| Ethnicity | Ahinkorah et al., 2021; Asa et al., 2017; Avogo, 2008; Campbell, 1994; CampbellE K, 1993; Ewemooje et al., 2020; Fayehun et al., 2020; Isiugo-abanihe, 1997; Kouame & Schellekens, 2002; Modile, 2017; Moultrie et al., 2001; Muvandi, 2003; Obembe et al., 2018; Omariba, 2006; Owoo et al., 2015; Perkins, 1997; Poukouta & Fapohunda, 1997; Takyi, 1993; Uchudi, 1999 |  | Ettyang, 1997; Staveteig, 2011 |
| Spouse influence | Angawa, 1997; Bolten & Marcantonio, 2021; Caplan, 1995; DeRose et al., 2002; Kahansim et al., 2013;Kahsay et al., 2018; Randall & LeGrand, 2003; Sahleyesus et al., 2009; Sinai et al., 2019; Telake, 2006; Teye, 2013; Towriss, 2014; Towriss et al., 2020; Ukaegbu, 2013; Upadhyay & Karasek, 2012; Vohra, 2014 | Novignon J. et al., 2019 | Reynar, 2000 |
| **Contemporary Disrupters** |  |  |  |
| Education | DeRose et al., 2002b; Dibaba, 2009; Ewemooje et al., 2020; Mahy, 2000;Matovu J.K.B. et al., 2017; McGinn, 2004; J. N. Nwogu et al., 2021; Sathiya Susuman A. et al., 2014; F. Tadesse & Headey, 2010 | Adewole et al., 2020; Agadjanian, 1995, 1998; Ahinkorah et al., 2020, 2021; Akeju et al., 2021; Akinyoade, 1999; Ambel, 2007; Amoateng, 1992; Angawa, 1997; Anonymous, 2016; Asa et al., 2017; Atake & Gnakou Ali, 2019; Avogo, 2008; Babalola et al., 2018; Banda, 1990; Bankole, 1995; Basu, 2002;Bauer et al., 2006; Baylies C., 2000; Behrman, 2015; Benefo, 1990; Benefo & Pillai, 2005; Bietsch, 2015; Capo-Chichi, 1999; Channon & Harper, 2019; Cannonier & Mocan, 2018;Cottrell, 2010; Derose & Ezeh, 2005; Dodoo, 1992;Dow Jr. et al., 1997; Durevall & Lindskog, 2008; Ekouevi, 1992; Elleamoh & Dake, 2019; Ettyang, 1997; FAIRLAMB & NIEUWOUDT, 1991; Fosu et al., 2013; Gasafari, 2006; Green, 1997; Heinz & Roth, 2019; Hollos M & Larsen U, 2004; Isiugo-Abanihe, 1994a; C. Izugbara et al., 2010; Joekes, 1994; Kahansim et al., 2013; (Keats, 2012); (Kebede et al., 2019)Kebede et al., 2021; Kiriti & Tisdell, 2005; Kodzi, 2009; Lemessa & Wencheko, 2014; Levin, 2001; Mahmud, 2017; Mashara, 2016; Mesfin, 2002; Modile, 2017; Muhoza, 2019; Mukasa, 2015; Mulenga et al., 2018; Novignon J. et al., 2019; Nwogu, 2006; Obembe et al., 2018; Obiero, 1998; O. C. Odimegwu, 1994; Okafor et al., 2021; Oladosu, 1999; Olalekan, 2013; Olenick, 1998;Osili & Long, 2008; Owoo et al., 2015; Oyediran & Isiugo-Abanihe, 2002; Perkins, 1997; Pierotti, 2013; Poukouta & Fapohunda, 1997; Robilliard, 2020; Rutayisire et al., 2013; Sahleyesus et al., 2009; Saila-Ngita et al., 2003; Sam et al., 2005; Shapiro, 2018; Shapiro & Tenikue, 2017 Snow et al., 2013; Solanke, 2017; I. S. Speizer, 1995; Staveteig, 2011; B. Tadesse & Asefa, 2002; Takyi, 1993; Telake, 2006; Teye, 2013; Thiede et al., 2020; Thomas, 2013; Togunde & Newman, 2005; Uchudi, 1999; Upadhyay & Karasek, 2012; Westoff & Bietsch, 2015; Yeatman et al., 2013 | Agadjanian & Prata, 2002; Ezra, 1997; Isiugo-Abanihe, 1994b; Musalia, 2000; Suda, 1997; Ukaegbu, 2013; Woldemicael, 2009 |
| Economy/Costs |  | Abdi et al., 2021; Adewole et al., 2020; Agadjanian, 2005; Akinyoade, 1999; Angawa, 1997; Banda, 1990; Baylies C., 2000; Bekele, 2004; Bietsch, 2015; Calvès & Meekers, 1999; CampbellE K, 1993; Capo-Chichi, 1999; Dahl-Jorgensen, 1990; DeRose et al., 2002; Ekouevi, 1992; Ezra, 1997; Garver, 2017; Green, 1997; Grieser et al., 2001; Gwako, 1997; Hayford & Agadjanian, 2019; Heinz & Roth, 2019; Hollos & Larsen, 1992, 1997; L. D. G. Ibisomi, 2008; L. Ibisomi & Odimegwu, 2011; Ibrahim, 2020, 2020; Imo et al., 2014; Izugbara & Ezeh, 2010; A. M. Jensen, 1991; A.-M. Jensen, 2015; Kahansim et al., 2013; LeGrand et al., 2003; Levin, 2001; Lloyd, 1993; Mahy, 2000; Mathur et al., 2016; Mjaaland T., 2014; Mosha et al., 2013; Musalia, 2000; Odusola & and others, 1998; Pierotti, 2013; Randall & LeGrand, 2003; Sahleyesus et al., 2009; Sam et al., 2005; Shreffler & Dodoo, 2009; Sinai et al., 2019; Smith, 1999, 2004; Staveteig, 2011; Suda, 1997; Swartz et al., 2018; Telake, 2006; Teye, 2013; Togunde & Newman, 2005; Towriss, 2014; Towriss et al., 2020; Van de Walle F. & Maiga M., 1991; Vohra, 2014; Wilson, 1998; Woldesenbet, 2010 | Kannae & Pendleton, 1994 |
| Socioeconomic status | Asa et al., 2017; Caplan, 1995; Dim, 2018; Hayford & Agadjanian, 2017; Kiriti & Tisdell, 2005; Kodzi et al., 2012; Lemessa & Wencheko, 2014; McGinn, 2004; B. Tadesse & Asefa, 2002; Towriss, 2014; Woldesenbet, 2010 | Adebowale & Palamuleni, 2015; Adewole et al., 2020; Ahinkorah et al., 2021; Akeju et al., 2021; Ambel, 2007; Angawa, 1997; Asuming, 2013; Babalola et al., 2018; Benefo, 1990; Bimha & Chadwick, 2016; Campbell, 1994; Capo-Chichi, 1999; Capurchande et al., 2017; Dibaba, 2009; Durevall & Lindskog, 2008; FAIRLAMB & NIEUWOUDT, 1991; Fosu et al., 2013; Garver, 2018; Green, 1997; Hollos M & Larsen U, 2004; Kebede et al., 2021; Kodzi, 2009; Mahmud, 2017; (Mjaaland T., 2014)Modile, 2017; Odusina, 2017; Owoo et al., 2015; Poukouta & Fapohunda, 1997; Robilliard, 2020; Snow et al., 2013; Togunde & Newman, 2005; Upadhyay & Karasek, 2012; Westoff & Bietsch, 2015; Woldemicael, 2009 | Avogo, 2008; Ettyang, 1997; I. S. Speizer, 1995; F. Tadesse & Headey, 2010 |
| Family planning |  | Ahinkorah et al., 2020; Akinyoade, 1999; Angawa, 1997; Avogo, 2008; Bekele, 2004; Benefo & Pillai, 2005;Bhargava A., 2007; Dibaba, 2009; Dow Jr. et al., 1997; Du Plessis, 1996; Ettyang, 1997;Gwako, 1997b; Green, 1997; Isiugo-Abanihe, 1994a; Isiugo-abanihe, 1997; Madhavan & Bledsoe, 2001; Matovu J.K.B. et al., 2017; Modile, 2017; Muvandi, 2003; Nwogu, 2006; Odusina, 2017; Odusina et al., 2020; Odusola & and others, 1998; Owoo et al., 2015; Owoo & Lambon-Quayefio, 2021; Oyediran, 2006; Oyediran & Isiugo-Abanihe, 2002; Poukouta & Fapohunda, 1997; Reynar, 2000;Rutayisire et al., 2013; Sahleyesus et al., 2009; Shreffler & Dodoo, 2009; Smith, 1999, 2004; Telake, 2006; Woldemicael, 2009 | Sathiya Susuman A. et al., 2014; Takyi, 1993; Umoh et al., 2012; Van de Walle F. & Maiga M., 1991 |
| Employment | Ewemooje et al., 2020; Mashara, 2016; Musalia, 2000; Takyi, 1993 | Acsadi & Johnson-Acsadi, 1990;Agadjanian, 1998; Ahinkorah et al., 2021; Amoateng, 1992; Angawa, 1997;Anonymous, 2016; Avogo, 2008; Babalola et al., 2018; Ewemooje et al., 2020; Gwako, 1997; Isiugo-abanihe, 1997;Mukasa, 2015; Nwakeze, 2007; Nwogu, 2006; C. O. Odimegwu et al., 2018; Odusina, 2017; Omariba, 2006;Owoo et al., 2015; Poukouta & Fapohunda, 1997; Robilliard, 2020;Togunde & Newman, 2005; Towriss et al., 2020;Uchudi, 1999; Uzobo & Odubo, 2016;Woldemicael, 2009 | Hussain, 1991; Kiriti & Tisdell, 2005; Lemessa & Wencheko, 2014; Novignon J. et al., 2019; I. S. Speizer, 1995 |
| Status of women | Angawa, 1997; Karp C. et al., 2020; Kirumira, 1996; LeVine & LeVine, 1998; Lloyd, 1993; Madhavan, 1998; Mukasa, 2015; Owoo et al., 2015; Staveteig, 2011 | Adebowale & Palamuleni, 2015; Adewole et al., 2020; Ahinkorah et al., 2020; Ambel, 2007;Atake & Gnakou Ali, 2019; Babalola et al., 2018; Baynes et al., 2020; Benefo, 1990; Beyeza-Kashesya et al., 2010; Bimha & Chadwick, 2016; FAIRLAMB & NIEUWOUDT, 1991; Gwako, 1997; Ibrahim, 2020a;Isiugo-Abanihe, 1994a; Madhavan & Bledsoe, 2001; Novignon J. et al., 2019; Nwakeze, 2007; Robilliard, 2020; Solanke, 2017; Towriss et al., 2020; Uchudi, 1999; Upadhyay & Karasek, 2012;Westoff & Bietsch, 2015 | Herman et al., 2011; Moultrie et al., 2001 |
| Spousal joint decision-making | Kodzi, 2009; Kodzi et al., 2012; Oyediran, 2006 | Asa et al., 2017; Avogo, 2008; Baynes et al., 2020; Derose & Ezeh, 2005; Dow Jr. et al., 1997; Ekouevi, 1992; Isiugo-Abanihe, 1994b, 1994a; Isiugo-abanihe, 1997; C. O. Izugbara & Ezeh, 2010; Muvandi, 2003; Odusina, 2017; Odusola & and others, 1998; Oladosu, 1999; Pierotti, 2013; Poukouta & Fapohunda, 1997; I. S. Speizer, 1995; Woldemicael, 2009 | Kahansim et al., 2013; Towriss, 2014 |
| HIV/AIDS epidemic | Hayford et al., 2012; Hayford & Agadjanian, 2017 | Cottrell, 2010; Durevall & Lindskog, 2008; Kirumira, 1996; Mahmud, 2017; Mahy, 2000; Mathur et al., 2016; Sahleyesus et al., 2009; Staveteig, 2011; Telake, 2006; Trinitapoli & Yeatman, 2011 | Baylies C., 2000; Kurth et al., 2015; Moyo & Mbizvo, 2004 |
| Mass media | Adebowale & Palamuleni, 2015; Ahinkorah et al., 2021; Hayford & Agadjanian, 2012; Lemessa & Wencheko, 2014; Mahy, 2000; Oyediran, 2006 | Avogo, 2008;Babalola et al., 2018;Bankole et al., 1996; Dibaba, 2009; Modile, 2017; Muvandi, 2003;Oyediran & Isiugo-Abanihe, 2002; Perkins, 1997; Smith, 2004; Westoff & Rodriguez, 1995;Westoff & Bietsch, 2015 | Sathiya Susuman A. et al., 2014 |
| Environmental factors |  | Baylies C., 2000; Bolten & Marcantonio, 2021; DiClemente et al., 2021; Eissler et al., 2019; Ezra, 1997, 2001; Garver, 2018; Joekes, 1994; Mjaaland T., 2014; Shreffler & Dodoo, 2009 | Hussain, 1991 |
| Conflict/civil unrest/violence | Rutayisire et al., 2013; Staveteig, 2011 | Agadjanian & Prata, 2002; McGinn, 2004; Mukasa, 2015; Thiede et al., 2020 |  |
| Legacy of colonialism/modernity |  | Capo-chichi & Juarez, 2001; LeVine & LeVine, 1998 |  |
| Migration | Hayford & Agadjanian, 2012; Hussain, 1991; McGinn, 2004; Smith, 2004 |  |  |
| Women’s Health Concerns |  | Baynes et al., 2020; Hayford & Agadjanian, 2019; A.-M. Jensen, 2017; Kodzi et al., 2012; Mairiga et al., 2010; Sinai et al., 2019; Towriss et al., 2020; Van de Walle F. & Maiga M., 1991; Wilson, 1998 |  |

**REFERENCES**

Abdi, B., Okal, J., Serour, G., & Temmerman, M. (2021). Muslim men’s perceptions and attitudes on family planning: A qualitative study in Wajir and Lamu counties in Kenya. *Sexual and Reproductive Health Matters*, *29*(1). https://doi.org/10.1080/26410397.2021.1893890

Acsadi, G. T. F., & Johnson-Acsadi, G. (1990). Demand for children and for childspacing. In G. T. F. Acsadi, G. Johnson-Acsadi, & R. A. Bulatao (Eds.), *Population growth and reproduction in sub-Saharan Africa: Technical analyses of fertility and its consequences.* (pp. 155–185). https://www.scopus.com/inward/record.uri?eid=2-s2.0-0025621881&partnerID=40&md5=a5752d6470461a2d55c35173a2157f32

Adebimpe, W. O., Asekun-Olarinmoye, E., Bamidele, J. O., & Abodunrin, O. (2011). A comparative study of socio-demographic determinants and fertility pattern among women in rural and urban communities in Southwestern Nigeria. *Continental Journal of Medical Research*, *5*(1), 32–40.

Adebowale, S. A., & Palamuleni, M. E. (2015). Influence of gender preference and sex composition of surviving children on childbearing intention among high fertility married women in stable union in Malawi. *African Health Sciences*, *15*(1), 150–160. https://doi.org/10.4314/ahs.v15i1.21

Adewole, O. G., Omotoso, K. O., & Asa, S. S. (2020). Contextual and Social Factors Influencing Male Fertility in Nigeria. *International Quarterly of Community Health Education*, *40*(3), 247–260. https://doi.org/10.1177/0272684X19875022

Adler, D. H., Abar, B., Bennie, T., Sadeghi, R., & Bekker, L.-G. (2017). Childbearing intentions among sexually active HIV-infected and HIV-uninfected female adolescents in South Africa. *Journal of AIDS and HIV Research (Online)*, *9*(7), 159–163. https://doi.org/10.5897/JAHR2017.0432

Adongo, P. B., Phillips, J. F., Kajihara, B., Fayorsey, C., Debpuur, C., & Binka, F. N. (1997). Cultural factors constraining the introduction of family planning among the Kassena-Nankana of Northern Ghana. *Social Science and Medicine*, *45*(12), 1789–1804. Scopus. https://doi.org/10.1016/S0277-9536(97)00110-X

Agadjanian, V. (1995). Fertility and society in Maputo, Mozambique [Ph.D., University of Southern California]. In *ProQuest Dissertations and Theses* (304225432). ProQuest Dissertations & Theses Global. http://ezproxy.cul.columbia.edu/login?url=https://search.proquest.com/docview/304225432?accountid=10226

Agadjanian, V. (1998). Economic security, informational resources, and women’s reproductive choices in urban Mozambique. *Social Biology*, *45*(1/2), 60. a9h. https://doi.org/10.1080/19485565.1998.9988964

Agadjanian, V. (2005). Fraught with Ambivalence: Reproductive Intentions and Contraceptive Choices in a Sub-Saharan Fertility Transition. *Population Research & Policy Review*, *24*(6), 617–645. sih. https://doi.org/10.1007/s11113-005-5096-8

Agadjanian, V., & Prata, N. (2002). War, peace, and fertility in Angola. *Demography*, *39*(2), 215–231. a9h. https://doi.org/10.1353/dem.2002.0013

Ahinkorah, B. O., Seidu, A. A., Armah-Ansah, E. K., Budu, E., Ameyaw, E. K., Agbaglo, E., & Yaya, S. (2020). Drivers of desire for more children among childbearing women in sub-Saharan Africa: Implications for fertility control. *BMC Pregnancy and Childbirth*, *20*(1), 778–778. https://doi.org/10.1186/s12884-020-03470-1

Ahinkorah, B. O., Seidu, A.-A., Armah-Ansah, E. K., Ameyaw, E. K., Budu, E., & Yaya, S. (2021). Socio-economic and demographic factors associated with fertility preferences among women of reproductive age in Ghana: Evidence from the 2014 Demographic and Health Survey. *Reproductive Health*, *18*(1), 2–2. https://doi.org/10.1186/s12978-020-01057-9

Akeju, K., Owoeye, T., Ayeni, R., & Jegede, L. (2021). Variations in desired fertility preferences among young and older women in nigeria: Evidence from demographic health survey 2018. *Open Public Health Journal*, *14*(1), 84–93. https://doi.org/10.2174/1874944502114010084

Akinyoade, Y. (1999). A comparative study of fertility preference in northwest and southwest Nigeria. *IFE Psychologia: An International Journal*, *7*(1), 96–122. psyh. https://doi.org/10.4314/ifep.v7i1.23548

Althaus, F. (1997). Comorian women delay marriage and first birth, but fertility... *International Family Planning Perspectives*, *23*(4), 187. a9h.

Ambel, A. A. (2007). Essays on intrahousehold allocation and the family: Fertility, child education, and nutrition [Ph.D., Western Michigan University]. In *ProQuest Dissertations and Theses* (304813611). ProQuest Dissertations & Theses Global. http://ezproxy.cul.columbia.edu/login?url=https://search.proquest.com/docview/304813611?accountid=10226

Amin, R., Chowdhury, J., & Hill, R. B. (1992). Socioeconomic differentials in contraceptive use and desire for more children in Greater Freetown, Sierra Leone. *International Family Planning Perspectives*, *18*(1), 24–26. Scopus. https://doi.org/10.2307/2133453

Amoateng, A. Y. (1992). Socio-cultural organization and fertility attitudes and behaviour of wives in Ezibeleni Township (Transkei). *Die Suid-Afrikaanse Tydskrif Vir Sosiologie. The South African Journal of Sociology*, *23*(4), 118–124. https://doi.org/10.1080/02580144.1992.10429866

Ampofo, A. A. (2002). Does women’s education matter in childbearing decision making? A case study from urban Ghana. *Ghana Studies*, *5*, 123–157. awn.

Ampofo, A. A., Okyerefo, M. P. K., & Pervarah, M. (2009). PHALLIC COMPETENCE: FATHERHOOD AND THE MAKING OF MEN IN GHANA. *Culture, Society and Masculinities*, *1*(1), 59–78. Sociological Abstracts. https://doi.org/10.3149/csm.0101.59

Angawa, F. O. P. (1997). Fertility dynamics and its relationship to child survival in Siaya district (BL) [Ph.D., University of Newcastle Upon Tyne (United Kingdom)]. In *PQDT - UK & Ireland* (301532513). ProQuest Dissertations & Theses Global. http://ezproxy.cul.columbia.edu/login?url=https://search.proquest.com/docview/301532513?accountid=10226

Anonymous. (2016). Demographic dividend In Africa: Prospect, opportunities and challenges. 7th African Population Conference of the Union for African Population Studies, Pretoria, South Africa, 30 November-4 December 2015. *African Population Studies*, *30*(2, Suppl.), 2341–3015.

Asa, S. S., Kupoluyi, J. A., Oyinloye, B. O., & Titilayo, A. (2017). Multivariate Analysis of Household Decision Making, Contraceptive Use and Fertility Behaviour among Ever-Married Men in Nigeria. *African Research Review*, *11*(4), 74–89. awn.

Atake, E.-H., & Gnakou Ali, P. (2019). Women’s empowerment and fertility preferences in high fertility countries in Sub-Saharan Africa. *BMC Women’s Health*, *19*(1), N.PAG-N.PAG. rzh. https://doi.org/10.1186/s12905-019-0747-9

Avogo, W. A. (2008). Social diffusion and fertility processes in Sub -Saharan Africa: Longitudinal evidence from Ghana [Ph.D., Arizona State University]. In *ProQuest Dissertations and Theses* (304686913). Sociological Abstracts. http://ezproxy.cul.columbia.edu/login?url=https://search.proquest.com/docview/304686913?accountid=10226

Ayoub, A. S. (2003). An economic analysis of women’s schooling on fertility and contraceptive use in developing countries: A case study of Tanzania [M.A., University of Nevada, Las Vegas]. In *ProQuest Dissertations and Theses* (305272967). ProQuest Dissertations & Theses Global. http://ezproxy.cul.columbia.edu/login?url=https://search.proquest.com/docview/305272967?accountid=10226

Babalola, S., Akinyemi, J. O., & Odimegwu, C. O. (2018). Changes in demand for children between 2003 and 2013 in Nigeria: Evidence from survey data. *Population Horizons*, *15*(1), 16–29. https://doi.org/10.2478/pophzn-2018-0006

Bachan, L. K. (2015). Safety Nets and Social Reproduction: Three Essays on Child Fostering in Sub-Saharan Africa in the Era of AIDS [Ph.D., The Pennsylvania State University]. In *ProQuest Dissertations and Theses* (2250413936). ProQuest Dissertations & Theses Global. http://ezproxy.cul.columbia.edu/login?url=https://search.proquest.com/docview/2250413936?accountid=10226

Banda, J. (1990). Value of children, well-being, and family size in Zambia. *Dissertation Abstracts International. A, Humanities and Social Sciences*, *50*(8), p.2661.

Bankole, A. (1995). Desired Fertility and Fertility Behaviour among the Yoruba of Nigeria: A Study of Couple Preferences and Subsequent Fertility. *Population Studies*, *49*(2), 317–328. Scopus. https://doi.org/10.1080/0032472031000148536

Bankole, A., Rodríguez, G., & Westoff, C. F. (1996). Mass media messages and reproductive behaviour in Nigeria. *Journal of Biosocial Science*, *28*(2), 227–239. https://doi.org/10.1017/s0021932000022264

Bankole, A., & Singh, S. (1998). Couples’ Fertility and Contraceptive Decision-Making in Developing Countries: Hearing the Man’s Voice. *International Family Planning Perspectives*, *24*(1), 15–24. Scopus. https://doi.org/10.2307/2991915

Bankole, A., Singh, S., Hussain, R., & Wulf, D. (2004). The sexual, marital and fathering behavior of men in Sub-Saharan Africa. *Etude de La Population Africaine*, *19*(2), 21–40. Scopus.

Barden-O’Fallon, J. (2005). Unmet Fertility Expectations and the Perception of Fertility Problems in a Malawian Village. *African Journal of Reproductive Health*, *9*(2), 14. https://doi.org/10.2307/3583459

Baschieri, A., Cleland, J., Floyd, S., Dube, A., Msona, A., Molesworth, A., Glynn, J. R., & French, N. (2013). REPRODUCTIVE PREFERENCES AND CONTRACEPTIVE USE: A COMPARISON OF MONOGAMOUS AND POLYGAMOUS COUPLES IN NORTHERN MALAWI. *Journal of Biosocial Science*, *45*(2), 145–166. https://doi.org/10.1017/S0021932012000569

Basu, A. M. (2002). Why does Education Lead to Lower Fertility? A Critical Review of Some of the Possibilities. *World Development*, *30*(10), 1779–1790. https://doi.org/10.1016/S0305-750X(02)00072-4

Bauer, M., Chytilová, J., & Streblov, P. (2006). *Effects of Education on Determinants of High Desired Fertility: Evidence from Ugandan Villages* (Working Paper No. 239; Institute of Economic Studies Working Paper). Institute of Economic Studies, Charles University.

Bawah, A. A., Akweongo, P., Simmons, R., & Phillips, J. F. (1999). Women’s fears and men’s anxieties: The impact of family planning on gender relations in northern Ghana. *Studies in Family Planning*, *30*(1), 54–66. https://doi.org/10.1111/j.1728-4465.1999.00054.x

Baylies C. (2000). The impact of HIV on family size preference in Zambia. *Reproductive Health Matters*, *8*(15), 77–86. Embase. https://doi.org/10.1016/S0968-8080(00)90008-9

Baynes, C., Yegon, E., Lusiola, G., Achola, J., & Kahando, R. (2020). Post-abortion fertility desires, contraceptive uptake and unmet need for family planning: Voices of post-abortion care clients in Tanzania. *Journal of Biosocial Science*, 1–16. https://doi.org/10.1017/S0021932020000607

Behrman, J. A. (2015). Does Schooling Affect Women’s Desired Fertility? Evidence From Malawi, Uganda, and Ethiopia. *Demography*, *52*(3), 787–809. rzh. https://doi.org/10.1007/s13524-015-0392-3

Bekele, D. (2004). Linkages among schooling, literacy, and fertility in Amhara region of Ethiopia [Ed.D., Harvard University]. In *ProQuest Dissertations and Theses* (305191691). ProQuest Dissertations & Theses Global. http://ezproxy.cul.columbia.edu/login?url=https://search.proquest.com/docview/305191691?accountid=10226

Benefo, K. D. (1990). The determinants of family size preferences and traditional child spacing practices in West Africa [Ph.D., University of Michigan]. In *ProQuest Dissertations and Theses* (303840823). ProQuest Dissertations & Theses Global. http://ezproxy.cul.columbia.edu/login?url=https://search.proquest.com/docview/303840823?accountid=10226

Benefo, K. D., & Pillai, V. K. (2005). The Reproductive Effects of Family Planning Programs in Rural Ghana: Analysis by Gender. *Journal of Asian and African Studies*, *40*(6), 463–477. a9h. https://doi.org/10.1177/0021909605059516

Berhanu, B. (1994). Fertility and contraceptive use in rural Dalle, Southern Ethiopia. *The Ethiopian Journal of Health Development*, *8*(1), 11–21. awn.

Beyeza-Kashesya, J., Neema, S., Ekstrom, A. M., Kaharuza, F., Mirembe, F., & Kulane, A. (2010). “Not a Boy, Not a Child”: A Qualitative Study on Young People’s Views on Childbearing in Uganda. *African Journal of Reproductive Health*, *14*(1), 71–81. GenderWatch.

Bhargava A. (2007). Desired family size, family planning and fertility in Ethiopia. *Journal of Biosocial Science*, *39*(3), 367–381. Embase. https://doi.org/10.1017/S0021932006001593

Bietsch, K. E. (2015). Men and contraception in sub-Saharan Africa [Ph.D., Princeton University]. In *ProQuest Dissertations and Theses* (1702157713). ProQuest Dissertations & Theses Global. http://ezproxy.cul.columbia.edu/login?url=https://search.proquest.com/docview/1702157713?accountid=10226

Bimha, P. Z. J., & Chadwick, R. (2016). Making the childfree choice: Perspectives of women living in South Africa. *JOURNAL OF PSYCHOLOGY IN AFRICA*, *26*(5), 449–456. https://doi.org/10.1080/14330237.2016.1208952

Bledsoe, C. (1990). Transformations in Sub-Saharan African Marriage and Fertility. *The ANNALS of the American Academy of Political and Social Science*, *510*(1), 115–125. https://doi.org/10.1177/0002716290510001009

Bolten, C. E., & Marcantonio, R. (2021). The paradox of planning: Agriculture, schooling, and the unresolvable uncertainty of ideal family size in Rural Sierra Leone. *African Studies Review*, 1–22. https://doi.org/10.1017/asr.2020.96

Bougma, M., Adjiwanou, V., & Kobiané, J.-F. (2021). Does sex composition of offspring relate to fertility in urban areas? The case of Ouagadougou, Burkina Faso. *Journal of Biosocial Science*, 1–16. https://doi.org/10.1017/S0021932021000031

Burgess, S., Kra, K. W., Allen, M., Vandermark, J., & Turke, S. (2020). Aspiring to establish a family: Reproductive health among young unmarried women in Abidjan, Côte d’Ivoire. *Culture, Health & Sexuality*, 1–16. https://doi.org/10.1080/13691058.2020.1826580

Calvès, A.-E., & Meekers, D. (1999). The advantages of having many children for women in formal and informal unions in Cameroon. *Journal of Comparative Family Studies*, *30*(4), 617–639. psyh.

Campbell, E. K. (1994). Fertility, family size preferences and future fertility prospects of men in the Western area of Sierra Leone. *Journal of Biosocial Science*, *26*(2), 273–277. psyh. https://doi.org/10.1017/S0021932000021301

Campbell, E. K., & Campbell, P. G. (1997). Family size and sex preferences and eventual fertility in Botswana. *Journal of Biosocial Science*, *29*(2), 191–204. psyh. https://doi.org/10.1017/S0021932097001910

CampbellE K. (1993). Family size preferences of men in the western area of Sierra Leone. Method and determinants. *Genus*, *49*(1–2), 181–199. awn.

Cannonier, C., & Mocan, N. (2018). THE IMPACT OF EDUCATION ON WOMEN’S PREFERENCES FOR GENDER EQUALITY: EVIDENCE FROM SIERRA LEONE. *JOURNAL OF DEMOGRAPHIC ECONOMICS*, *84*(1, SI), 3–40. https://doi.org/10.1017/dem.2016.12

Caplan, P. [Ann P. C. (1995). “Children are our wealth and we want them”: A difficult pregnancy on northern Mafia Island, Tanzania. In Deborah Fahy Bryceson (Ed.), *Women wielding the hoe* (pp. 131–149). Berg; awn. https://proxy.lib.ohio-state.edu/login?url=http://search.ebscohost.com/login.aspx?direct=true&db=awn&AN=LEIDEN-149007493&site=ehost-live

Capo-Chichi, P. V. A. (1999). Fertility transition in benin: New reproductive patterns or traditional behaviours? [Ph.D., University of London, London School of Hygiene and Tropical Medicine (United Kingdom)]. In *PQDT - UK & Ireland* (301638999). ProQuest Dissertations & Theses Global. http://ezproxy.cul.columbia.edu/login?url=https://search.proquest.com/docview/301638999?accountid=10226

Capo-chichi, V., & Juarez, F. (2001). Is fertility declining in Benin? *Studies in Family Planning*, *32*(1), 25–40. psyh. https://doi.org/10.1111/j.1728-4465.2001.00025.x

Capurchande, R., Coene, G., Roelens, K., & Meulemans, H. (2017). “If I have only two children and they die… who will take care of me?” -a qualitative study exploring knowledge, attitudes and practices about family planning among Mozambican female and male adults. *BMC Women’s Health*, *17*, 1–15. rzh. https://doi.org/10.1186/s12905-017-0419-6

Channon, M. D., & Harper, S. (2019). Educational differentials in the realisation of fertility intentions: Is sub-Saharan Africa different? *PLoS ONE*, *14*(7), 1–14. a9h. https://doi.org/10.1371/journal.pone.0219736

Cottrell, E. K. B. (2010). Fertility in an era of HIV/AIDS: The impact of HIV/AIDS on the reproductive preferences and behavior of adolescent women in Cape Town, South Africa [Ph.D., The University of Wisconsin - Madison]. In *ProQuest Dissertations and Theses* (822410618). ProQuest Dissertations & Theses Global. http://ezproxy.cul.columbia.edu/login?url=https://search.proquest.com/docview/822410618?accountid=10226

Dahl-Jorgensen, C. (1990). Fertility behaviour of peasants: The case of Endode. *Working Papers on Ethiopian Development, Ethiopia Research Programme, College of Arts and Science, University of Trondheim*, *5*, 113–128.

DeRose, L. F. (2007). Marriage Type and Relative Spousal Power in Ghana: Changing Effects of Monogamy During Early Fertility Decline. *Journal of Comparative Family Studies*, *38*(1), 125–141. a9h. https://doi.org/10.3138/jcfs.38.1.125

DeRose, L. F., Dodoo, F. N.-A., & Patil, V. (2002a). Fertility desires and perceptions of power in reproductive conflict in Ghana. *Gender & Society*, *16*(1), 53. sih. https://doi.org/10.1177/0891243202016001004

DeRose, L. F., Dodoo, F. N.-A., & Patil, V. (2002b). Schooling and Attitudes on Reproductive-Related Behavior in Ghana. *International Journal of Sociology of the Family*, *30*(1), 50–65. Sociological Abstracts.

Derose, L. F., & Ezeh, A. C. (2005). Men’s influence on the onset and progress of fertility decline in Ghana, 1988–98. *Population Studies*, *59*(2), 197–210. a9h. https://doi.org/10.1080/00324720500099496

Dibaba, Y. (2009). Factors influencing women’s intention to limit child bearing in Oromia, Ethiopia. *Ethiopian Journal of Health Development*, *23*(1), 28–33.

DiClemente, K., Grace, K., Kershaw, T., Bosco, E., & Humphries, D. (2021). Investigating the Relationship between Food Insecurity and Fertility Preferences in Tanzania. *Maternal and Child Health Journal*, *25*(2), 302–310. https://doi.org/10.1007/s10995-020-03022-1

Dim, E. E. (2018). Family structure and fertility behaviour among undergraduates of the distant learning institute in Lagos State, Nigeria. *Etude de La Population Africaine*, *32*(1). International Bibliography of the Social Sciences (IBSS). https://doi.org/10.11564/32-1-1145

Dodoo, F. N.-A. (1992). Female education, age, parity, and reproduction cessation in Ghana. *Biodemography and Social Biology*, *39*(1–2), 102–108. psyh. https://doi.org/10.1080/19485565.1992.9988807

Dodoo, F. N.-A. (1998). Marriage type and reproductive decisions: A comparative study in Sub-Saharan Africa. *Journal of Marriage & Family*, *60*(1), 232–242. ssa. https://doi.org/10.2307/353454

Dow Jr., T. E., Kekovole, J., & Archer, L. H. (1997). Wealth flow and fertility decline in rural Kenya, 1981-92: A reassessment of the evidence. *African Journal of Reproductive Health*, *1*(2), 41–66. Scopus. https://doi.org/10.2307/3583376

Du Plessis, G. (1996). Reproductive choice and motivation in South Africa, 1987-1989. *Southern African Journal of Demography*, *6*(1), 33–48. awn.

Durevall, D., & Lindskog, A. (2008). HIV/AIDS, Adult Mortality and Fertility: Evidence from Malawi. In *SIDA, Swedish International Development Cooperation Agency, Jan 2008, 54 pp.* (58768503; 2008-128731; p. 54p). SIDA, Swedish International Development Cooperation Agency; PAIS Index. http://ezproxy.cul.columbia.edu/login?url=https://search.proquest.com/docview/58768503?accountid=10226

Dybdahl, R., & Hundeide, K. (1998). Childhood in the Somali context: Mothers’ and children’s ideas about childhood and parenthood. *Psychology and Developing Societies*, *10*(2), 131–145. psyh. https://doi.org/10.1177/097133369801000203

Dyer, S., Mokoena, N., Maritz, J., & van der Spuy, Z. (2008). Motives for parenthood among couples attending a level 3 infertility clinic in the public health sector in South Africa. *Human Reproduction*, *23*(2), 352–357. https://doi.org/10.1093/humrep/dem279

Eissler, S., Thiede, B. C., & Strube, J. (2019). Climatic variability and changing reproductive goals in Sub-Saharan Africa. *Global Environmental Change*, *57*. Scopus. https://doi.org/10.1016/j.gloenvcha.2019.03.011

Ekouevi, K. A. (1992). Family and reproductive behavior in urban Togo [Ph.D., University of Pennsylvania]. In *ProQuest Dissertations and Theses* (303999265). ProQuest Dissertations & Theses Global. http://ezproxy.cul.columbia.edu/login?url=https://search.proquest.com/docview/303999265?accountid=10226

Elleamoh, G. E., & Dake, F. A. A. (2019). “Cementing” marriages through childbearing in subsequent unions: Insights into fertility differentials among first-time married and remarried women in Ghana. *PloS One*, *14*(10), e0222994. https://doi.org/10.1371/journal.pone.0222994

Ettyang, L. I. A. (1997). Exogenous covariates of desire for another child: The Kenyan experience [Ph.D., University of Southern California]. In *ProQuest Dissertations and Theses* (304370803). ProQuest Dissertations & Theses Global. http://ezproxy.cul.columbia.edu/login?url=https://search.proquest.com/docview/304370803?accountid=10226

Ewemooje, O. S., Biney, E., & Amoateng, A. Y. (2020). Determinants of fertility intentions among women of reproductive age in South Africa: Evidence from the 2016 demographic and health survey. *Journal of Population Research*. https://doi.org/10.1007/s12546-020-09246-w

Eyayou, Y., Berhane, Y., & Zerihun, L. (2004). Socio-cultural factors in decisions related to fertility in remotely located communities: The case of the Suri ethnic group. *Ethiopian Journal of Health Development*, *18*(3), 171–174.

Ezeh, A. C. (1997). Polygyny and reproductive behavior in Sub-Saharan Africa: A contextual analysis. *Demography*, *34*(3), 355–368. ssa. https://doi.org/10.2307/3038289

Ezra, M. (1997). Demographic responses to ecological degradation and food insecurity: Drought prone areas in Northern Ethiopia. In *Demographic responses to ecological degradation and food insecurity: Drought prone areas in Northern Ethiopia.* (p. 373 pp.).

Ezra, M. (2001). Demographic responses to environmental stress in the drought- and famine-prone areas of Northern Ethiopia. *International Journal of Population Geography*, *7*(4), 259–279. Scopus. https://doi.org/10.1002/ijpg.226

Fairlamb, C. D. (1990). Economic factors affecting human fertility in the developing areas of South Africa: A policy perspective. *Agrekon*, *29*(4), 284–289. Scopus. https://doi.org/10.1080/03031853.1990.9525113

Fairlamb, C. D., & Nieuwoudt, W. L. (1991). An economic analysis of family size decision making with reference to the developing areas of South Africa. *Development Southern Africa*, *8*(4), 513–520. Scopus. https://doi.org/10.1080/03768359108439611

Farmer, D. B., Berman, L., Ryan, G., Habumugisha, L., Basinga, P., Nutt, C., Kamali, F., Ngizwenayo, E., St Fleur, J., Niyigena, P., Ngabo, F., Farmer, P. E., & Rich, M. L. (2015). Motivations and Constraints to Family Planning: A Qualitative Study in Rwanda’s Southern Kayonza District. *Global Health Science and Practice*, *3*(2), 242–254. https://doi.org/10.9745/GHSP-D-14-00198

Fayehun, O., Sanuade, O. A., Ajayi, A. I., & Isiugo-Abanihe, U. (2020). Ethnicity, sex composition of living children, and unrealized fertility in Nigeria. *Population Studies*, 1–11. https://doi.org/10.1080/00324728.2020.1779333

Fitaw, Y., Berhane, Y., & Worku, A. (2004). Impact of child mortality and fertility preferences on fertility status in rural Ethiopia. *East African Medical Journal*, *81*(6), 300–306. Scopus. https://doi.org/10.4314/eamj.v81i6.9179

Fosu, M. O., Nyarko, P. R., & Anokye, M. (2013). The desire for last birth among Ghanaian women: The determinants. *Research on Humanities and Social Sciences*, *3*(22), 122–129.

Garver, S. (2017). Uncertainty and Fertility Preferences in Rural Malawi: Planning for Children When the Future Is Unknown [Ph.D., The Ohio State University]. In *ProQuest Dissertations and Theses* (2183919958). ProQuest Dissertations & Theses Global. http://ezproxy.cul.columbia.edu/login?url=https://search.proquest.com/docview/2183919958?accountid=10226

Garver, S. (2018). Navigating livelihood uncertainty: Prevailing wisdoms guiding fertility preferences in rural Malawi. *African Population Studies*, *32*(1), 3964–3973. sih. https://doi.org/10.11564/32-1-1162

Gasafari, W. (2006). *Communication about family planning and desired fertility among married people in Rwanda*. awn. https://proxy.lib.ohio-state.edu/login?url=http://search.ebscohost.com/login.aspx?direct=true&db=awn&AN=NX0174543&site=ehost-live

Geleta, D. (2018). Femininity, masculinity and family planning decision-making among married men and women in rural Ethiopia: A qualitative study. *Journal of African Studies and Development*, *10*(9), 124–133. https://doi.org/10.5897/JASD2018.0498

Green, S. R. (1997). *The roles of social interaction and male involvement in fertility decline in sub-Saharan Africa: Three essays* (304394735) [Ph.D., University of Pennsylvania]. ProQuest Dissertations & Theses Global. http://ezproxy.cul.columbia.edu/login?url=https://search.proquest.com/docview/304394735?accountid=10226

Grieser, M., Gittelsohn, J., Shankar, A. V., Koppenhaver, T., Legrand, T. K., Marindo, R., Mavhu, W. M., & Hill, K. (2001). Reproductive Decision Making and the HIV/AIDS Epidemic in Zimbabwe. *Journal of Southern African Studies*, *27*(2), 225–243. a9h. https://doi.org/10.1080/03057070120049949

Gwako, E. L. M. (1997a). Conjugal power in rural Kenya families: Its influence on women’s decisions about family size and family planning practices. *Sex Roles*, *36*(3–4), 127–147. Scopus. https://doi.org/10.1007/bf02766264

Gwako, E. L. M. (1997b). Married women’s ideal family size preferences and family planning practices: Evidence from rural... *Social Science Journal*, *34*(3), 369. sih. https://doi.org/10.1016/S0362-3319(97)90035-5

Harrison, A., & Montgomery, E. (2001). Life histories, reproductive histories: Rural South African women’s narratives of fertility, reproductive health and illness. *Journal of Southern African Studies*, *27*(2), 311–328. https://doi.org/10.1080/03057070120049994

Hayford, S. R., & Agadjanian, V. (2012). From desires to behavior: Moderating factors in a fertility transition. *Demographic Research*, *26*, 511–542. sih. https://doi.org/10.4054/DemRes.2012.26.20

Hayford, S. R., & Agadjanian, V. (2017). Determined to stop? Longitudinal analysis of the desire to have no more children in rural Mozambique. *Population Studies*, *71*(3), 329–344. a9h. https://doi.org/10.1080/00324728.2017.1334957

Hayford, S. R., & Agadjanian, V. (2019). Spacing, Stopping, or Postponing? Fertility Desires in a Sub-Saharan Setting. *Demography*, *56*(2), 573–594. a9h. https://doi.org/10.1007/s13524-018-0754-8

Hayford, S. R., Agadjanian, V., & Luz, L. (2012). Now or never: Perceived HIV status and fertility intentions in rural Mozambique. *Studies in Family Planning*, *43*(3), 191–199. a9h. https://doi.org/10.1111/j.1728-4465.2012.00317.x

Heinz, E. M., & Roth, L. M. (2019). As many as i can afford: Ideal family size in Contemporary Uganda. In *Reproductive Justice and Sexual Rights: Transnational Perspectives* (pp. 191–212). Scopus. https://www.scopus.com/inward/record.uri?eid=2-s2.0-85071504197&partnerID=40&md5=0cfcf15c1e35fdfeb42be472f30379d3

Herman, M., Hogan, D., Belachew, T., Tessema, F., Gebremariam, A., Lindstrom, D., Hadley, C., Jira, C., & Woldemichael, K. (2011). Better-Educated Youth as a Vanguard of Social Change? Adolescent Transitions to Later Marriage and Lower Fertility in Southwest Ethiopia. In C. Teller & A. Hailemariam (Eds.), *Demographic Transition and Development in Africa: The Unique Case of Ethiopia* (pp. 89–101). https://doi.org/10.1007/978-90-481-8918-2_5

Hollos, M., & Larsen, U. (1992). Fertility differentials among the Ijo in southern Nigeria: Does urban residence make a difference? *Social Science & Medicine*, *35*(9), 1199–1210. https://doi.org/10.1016/0277-9536(92)90232-F

Hollos, M., & Larsen, U. (1997). From Lineage to Conjugality: The Social Context of Fertility Decisions among the Pare of Northern Tanzania. *Social Science & Medicine*, *45*(3), 361–372. Sociological Abstracts. https://doi.org/10.1016/s0277-9536(96)00351-6

Hollos M & Larsen U. (2004). Which African men promote smaller families and why? Marital relations and fertility in a Pare community in Northern Tanzania. *Social Science & Medicine*, *58*(9), 1733–1749. a9h. https://doi.org/10.1016/S0277-9536(03)00365-4

Hough, C. A. (2008). RE/PRODUCING MOTHERS: STRUCTURE AND AGENCY IN GAMBIAN KANYALENG PERFORMANCES. *Ethnology*, *47*(4), 257–269.

Hussain, F. (1991). Landholding and human fertility in Rwanda [Ph.D., Michigan State University]. In *ProQuest Dissertations and Theses* (303950735). ProQuest Dissertations & Theses Global. http://ezproxy.cul.columbia.edu/login?url=https://search.proquest.com/docview/303950735?accountid=10226

Ibisomi, L. D. G. (2008). Fertility Transition in Nigeria: Exploring the Role of Desired Number of Children. *Etude de La Population Africaine/African Population Studies*, *23*(2), 207–222. Sociological Abstracts.

Ibisomi, L., & Odimegwu, C. (2011). Understanding resolution of differential fertility preferences among couples in Nigeria. *International Journal of Business and Social Science*, *2*(4), 98–105.

Ibrahim, F. M. (2020a). Current use of modern family planning and fertility intention among women farmers of reproductive age in Ido and Ona-ara local government areas of Ibadan, Nigeria. *Rural Sustainability Research*, *43*(388), 9–19. https://doi.org/10.2478/plua-2020-0002

Ibrahim, F. M. (2020b). “Everyone tries to avoid responsibility” The attenuating role of financial obligations in fertility change among Yorùbá farmers of southwestern Nigeria. *Demographic Research*, *43*, 745–778. https://doi.org/10.4054/DemRes.2020.43.26

Ibrahim, F. M. (2020c). RETHINKING THE AFRICAN VALUE OF HIGH FERTILITY: THE YORÙBÁ FARMERS’ EXAMPLE. *Modern Africa: Politics, History & Society*, *8*(1), 11–34. sih.

Ibrahim, F. M., & Arulogun, O. S. (2020). Posterity and population growth: Fertility intention among a cohort of Nigerian adolescents. *Journal of Population Research*, *37*(1), 25–52. sih. https://doi.org/10.1007/s12546-019-09230-z

Imo, C., Okoronkwo, E., & Ukoji, V. (2014). The Role of socioeconomic factors in fertility of Umuahia women in Abia State, Nigeria. *International Journal of Development and Management Review*, *9*(1), 227–247. awn.

Isiugo-Abanihe, U. C. (1994a). Reproductive motivation and family-size preferences among Nigerian men. *Studies in Family Planning*, *25*(3), 149–161. https://doi.org/10.2307/2137941

Isiugo-Abanihe, U. C. (1994b). The Socio-Cultural Context of High Fertility among Igbo Women. *International Sociology*, *9*(2), 237–258. Sociological Abstracts. https://doi.org/10.1177/026858094009002008

Isiugo-abanihe, U. C. (1997). Fertility preferences and contraceptive practice in Nigeria. *Annals of the Social Science Council of Nigeria*, *9*, 1–20. Scopus.

Izugbara, C., Ibisomi, L., Ezeh, A. C., & Mandara, M. (2010). Gendered interests and poor spousal contraceptive communication in Islamic northern Nigeria. *Journal of Family Planning and Reproductive Health Care*, *36*(4), 219–224. https://doi.org/10.1783/147118910793048494

Izugbara, C. O., & Ezeh, A. C. (2010). Women and high fertility in Islamic northern Nigeria. *Studies in Family Planning*, *41*(3), 193–204. a9h. https://doi.org/10.1111/j.1728-4465.2010.00243.x

Jensen, A. M. (1991). Economic change, marriage relations and fertility in a rural area of Kenya. In K. A. Stolen & M. Vaa (Eds.), *Gender and change in developing countries.* (pp. 67–89).

Jensen, A.-M. (2015). Poverty, gender and fertility in rural Kenya. *Forum for Development Studies*, *42*(2), 311–332. Scopus. https://doi.org/10.1080/08039410.2015.1025827

Jensen, A.-M. (2017). Comparing family changes in two rural areas of Kenya: Past legacies and present realities. *DEVELOPMENT SOUTHERN AFRICA*, *34*(6, SI), 787–801. https://doi.org/10.1080/0376835X.2017.1318701

Joekes, S. (1994). Children as a resource: Environmental degradation and fertility. *Focus on Gender*, *2*(2), 13–18. sih.

Kahansim, M. L., Hadejia, I. S., & Sambo, M. N. (2013). A Comparative Study of Factors Influencing Decisions on Desired Family Size among Married Men and Women in Bokkos, a Rural Local Government Area in Plateau State. *African Journal of Reproductive Health*, *17*(1), 149–157. rzh.

Kahsay, Z. H., Alemayehu, M., Medhanyie, A. A., & Mulugeta, A. (2018). Drivers to have more children in the pastoralist communities of Afar, Ethiopia: An explorative qualitative study. *Ethiopian Journal of Health Development*, *32*(Special Issue), 21–27.

Kalesanwo, O. O., & Adenuga Emmanuel, A. (2009). Assessment of adults’ opinion on the ideal family size and family well-being in Ogun state, Nigeria. *Academic Leadership*, *7*(3). Scopus. https://www.scopus.com/inward/record.uri?eid=2-s2.0-69549115137&partnerID=40&md5=5c6c899de6f3ad95e0467833f27acceb

Kannae, L., & Pendleton, B. F. (1994). Fertility Attitudes among Male Ghanaian Government Employees. *Journal of Asian and African Studies*, *29*(1–2), 65–76. Sociological Abstracts. https://doi.org/10.1177/002190969402900104

Karp C., Wood S.N., Galadanci H., Sebina Kibira S.P., Makumbi F., Omoluabi E., Shiferaw S., Seme A., Tsui A., & Moreau C. (2020). ‘I am the master key that opens and locks’: Presentation and application of a conceptual framework for women’s and girls’ empowerment in reproductive health. *Social Science and Medicine*, *258*((Karp C., celia.karp@jhu.edu; Wood S.N.; Tsui A.; Moreau C.) Department of Population, Family and Reproductive Health, Johns Hopkins Bloomberg School of Public Health, United States). Embase. https://doi.org/10.1016/j.socscimed.2020.113086

Keats, A. B. (2012). Essays on Development Economics: Occupational Choice in Rural Kenya: Using Subjective Expectations Data to Measure Credit and Insurance Constraints Challenges in Banking the Rural Poor: Evidence from Kenya’s Western Province Female Education, Fertility, and Child Health in Uganda [Ph.D., University of California, Los Angeles]. In *ProQuest Dissertations and Theses* (1022487739). ProQuest Dissertations & Theses Global. http://ezproxy.cul.columbia.edu/login?url=https://search.proquest.com/docview/1022487739?accountid=10226

Kebede, E., Goujon, A., & Lutz, W. (2019). Stalls in Africa’s fertility decline partly result from disruptions in female education. *Proceedings of the National Academy of Sciences*, *116*(8), 2891. https://doi.org/10.1073/pnas.1717288116

Kebede, E., Striessnig, E., & Goujon, A. (2021). The relative importance of women’s education on fertility desires in sub-Saharan Africa: A multilevel analysis. *Population Studies*, 1–20. https://doi.org/10.1080/00324728.2021.1892170

Kidman, R., & Anglewicz, P. (2014). Fertility Among Orphans in Rural Malawi: Challenging Common Assumptions About Risk and Mechanisms. *International Perspectives on Sexual & Reproductive Health*, *40*(4), 164–175. a9h. https://doi.org/10.1363/4016414

Kiriti, T. W., & Tisdell, C. (2005). Family size, economics and child gender preference: A case study in the Nyeri district of Kenya. *International Journal of Social Economics*, *32*(6), 492–509. https://doi.org/10.1108/03068290510596989

Kirumira, E. K. (1996). *Familial relationships and population dynamics in Uganda: A case study of fertility behavior in the Central Region* (pp. 263–263). Univ., Sociologisk inst; awn. https://proxy.lib.ohio-state.edu/login?url=http://search.ebscohost.com/login.aspx?direct=true&db=awn&AN=K-015370&site=ehost-live

Kodzi, I. A. (2009). *Three essays on the fertility preferences of rural Ghanaian women: A longitudinal perspective* (875175073) [Ph.D., The Pennsylvania State University]. GenderWatch. http://ezproxy.cul.columbia.edu/login?url=https://search.proquest.com/docview/875175073?accountid=10226

Kodzi, I. A., Casterline, J. B., & Aglobitse, P. (2010). The time dynamics of individual fertility preferences among rural Ghanaian women. *Studies in Family Planning*, *41*(1), 45–54. psyh. https://doi.org/10.1111/j.1728-4465.2010.00223.x

Kodzi, I. A., Johnson, D. R., & Casterline, J. B. (2012). To have or not to have another child: Life cycle, health and cost considerations of Ghanaian women. *Social Science & Medicine*, *74*(7), 966–972. a9h. https://doi.org/10.1016/j.socscimed.2011.12.035

Kouame, A., & Schellekens, J. (2002). Rural Development and Attitudes towards Family Size in Cote d’Ivoire. *Population*, *57*(2), 269–300. Sociological Abstracts. https://doi.org/10.2307/3246610

Kurth, A. E., Inwani, I., Wangombe, A., Nduati, R., Owuor, M., Njiri, F., Akinyi, P., Cherutich, P., Osoti, A., Kinuthia, J., Chhun, N., & Kiarie, J. (2015). The Gender Context of HIV Risk and Pregnancy Goals in Western Kenya. *East African Medical Journal*, *92*(4), 163–169.

LeGrand, T., Koppenhaver, T., Mondain, N., & Randall, S. (2003). Reassessing the Insurance Effect: A Qualitative Analysis of Fertility Behavior in Senegal and Zimbabwe. *Population & Development Review*, *29*(3), 375–403. a9h. https://doi.org/10.1111/j.1728-4457.2003.00375.x

Lemessa, R., & Wencheko, E. (2014). Factors affecting the intention of women to limit childbearing in rural Ethiopia. *Ethiopian Journal of Health Development*, *28*(2), 75–80.

Levin, E. C. (2001). Women’s childbearing decisions in Guinea: Life course perspectives and historical change. *Africa Today*, *47*(3/4), 62–81. ssa.

LeVine, R. A., & LeVine, S. (1998). Fertility and maturity in Africa: Gusii parents in middle adulthood. In R. A. Shweder (Ed.), *Welcome to middle age! : (And other cultural fictions).* (1998-07748-006; pp. 189–207). The University of Chicago Press; psyh. https://proxy.lib.ohio-state.edu/login?url=http://search.ebscohost.com/login.aspx?direct=true&db=psyh&AN=1998-07748-006&site=ehost-live

Lloyd, C. B. (1993). Family & gender issues for population policy. *Family & Gender Issues for Population Policy*. sih. https://proxy.lib.ohio-state.edu/login?url=http://search.ebscohost.com/login.aspx?direct=true&db=sih&AN=SN051939&site=ehost-live

Lowe, H., Kenny, L., Hassan, R., Bacchus, L. J., Njoroge, P., Dagadu, N. A., Hossain, M., & Cislaghi, B. (2021). “If she gets married when she is young, she will give birth to many kids”: A qualitative study of child marriage practices amongst nomadic pastoralist communities in Kenya. *Culture, Health & Sexuality*, 1–17. https://doi.org/10.1080/13691058.2021.1893821

Madhavan, S. (1998). Collaboration and conflict among women in rural Mali: Effects on fertility and child survival [Ph.D., University of Pennsylvania]. In *ProQuest Dissertations and Theses* (304475210). ProQuest Dissertations & Theses Global. http://ezproxy.cul.columbia.edu/login?url=https://search.proquest.com/docview/304475210?accountid=10226

Madhavan, S., & Bledsoe, C. H. (2001). The compound as a locus of fertility management: The case of The Gambia. *Culture, Health & Sexuality*, *3*(4), 451–468. a9h. https://doi.org/10.1080/13691050110074219

Mahmud, A. (2017). Behavioral or Biological: Taking a Closer Look at the Relationship Between HIV and Fertility. In Hoque, MN and Pecotte, B and McGehee, MA (Ed.), *APPLIED DEMOGRAPHY AND PUBLIC HEALTH IN THE 21ST CENTURY* (Vol. 8, pp. 361–380). https://doi.org/10.1007/978-3-319-43688-3_20

Mahy, M. I. (2000). *Perceptions of child mortality and their effects on fertility intentions in Zimbabwe* (304668647) [Dr.Sc., The Johns Hopkins University, School of Public Health and Hygiene]. ProQuest Dissertations & Theses Global. http://ezproxy.cul.columbia.edu/login?url=https://search.proquest.com/docview/304668647?accountid=10226

Mairiga, A. G., Kullima, A. A., Bako, B., & Kolo, M. A. (2010). Sociocultural factors influencing decision-making related to fertility among the Kanuri tribe of north-eastern Nigeria. *African Journal of Primary Health Care & Family Medicine*, *2*(1), 94. https://doi.org/10.4102/phcfm.v2i1.94

Mashara, J. N. (2016). Factors influencing fertility preferences of currently married men in Kenya. *African Population Studies*, *30*, 2431–2443. sih.

Mathur, S., Higgins, J. A., Thummalachetty, N., Rasmussen, M., Kelley, L., Nakyanjo, N., Nalugoda, F., & Santelli, J. S. (2016). Fatherhood, marriage and HIV risk among young men in rural Uganda. *Culture, Health & Sexuality*, *18*(5), 538–552. a9h. https://doi.org/10.1080/13691058.2015.1091508

Matovu J.K.B., Makumbi F., Wanyenze R.K., & Serwadda D. (2017). Determinants of fertility desire among married or cohabiting individuals in Rakai, Uganda: A cross-sectional study. *Reproductive Health*, *14*(1). Embase. https://doi.org/10.1186/s12978-016-0272-3

Mayer B & Trommsdorff G. (2010). Adolescents’ value of children and their intentions to have children: A cross-cultural and multilevel analysis. *Journal of Cross-Cultural Psychology*, *41*(5/6), 671–689. rzh. https://doi.org/10.1177/0022022110372195

McGinn, T. (2004). *The Effects of Conflict on Fertility Desires and Behavior in Rwanda* (1562267936) [Dr.P.H., Columbia University]. ProQuest Dissertations & Theses Global. http://ezproxy.cul.columbia.edu/login?url=https://search.proquest.com/docview/1562267936?accountid=10226

Mesfin, G. (2002). The role of men in fertility and family planning program in Tigray Region. *Ethiopian Journal of Health Development*, *16*(3), 247–255.

Milazzo, A. (2014). Son preference, fertility and family structure: Evidence from reproductive behavior among Nigerian women. *Policy Research Working Paper - World Bank*, *6869*, 44 pp.

Mjaaland T. (2014). Having fewer children makes it possible to educate them all: An ethnographic study of fertility decline in north-western Tigray, Ethiopia. *Reproductive Health Matters*, *22*(43), 104–112. Embase. https://doi.org/10.1016/S0968-8080(14)43768-6

Modile, A. (2017). Intergenerational Transmission of Fertility Intentions among Nigerian Women [M.A., University of Colorado at Boulder]. In *ProQuest Dissertations and Theses* (1978073628). ProQuest Dissertations & Theses Global. http://ezproxy.cul.columbia.edu/login?url=https://search.proquest.com/docview/1978073628?accountid=10226

Mosha, I., Ruben, R., & Kakoko, D. (2013). Family planning decisions, perceptions and gender dynamics among couples in Mwanza, Tanzania: A qualitative study. *BMC Public Health*, *13*, 523. https://doi.org/10.1186/1471-2458-13-523

Moultrie, T. A. (2001). Special issue on fertility in Southern Africa: Selected articles. *Journal of Southern African Studies*, *27*(2), 207–258. awn.

Moyo, W., & Mbizvo, M. T. (2004). Desire for a Future Pregnancy Among Women in Zimbabwe in Relation to Their Self-Perceived Risk of HIV Infection, Child Mortality, and Spontaneous Abortion. *AIDS and Behavior*, *8*(1), 9–15. Sociology Database. https://doi.org/10.1023/B:AIBE.0000017521.26426.9d

Muhoza, D. N. (2019). The heterogeneous effects of socioeconomic and cultural factors on fertility preferences: Evidence from Rwanda and Kenya. *Journal of Population Research*, *36*(4), 347–363. sih. https://doi.org/10.1007/s12546-019-09227-8

Mukasa, N. (2015). Violent Conflict and Forced Displacement A Comparison of Family Size Dynamics and Women’s Power in Uganda [Ph.D., Universidad de Deusto (Spain)]. In *PQDT - Global* (2039579714). ProQuest Dissertations & Theses Global. http://ezproxy.cul.columbia.edu/login?url=https://search.proquest.com/docview/2039579714?accountid=10226

Mulenga, J., Mulenga, M. C., Bwalya, B. B., & Ngongola-Reinke, C. (2018). Too young to be a wife! Analysis of the factors influencing child marriages and its influence on the preferred number of children among women in Zambia. *Etude de La Population Africaine*, *32*(2), 4319–4331. International Bibliography of the Social Sciences (IBSS). https://doi.org/10.11564/32-2-1210

Musalia, J. M. (2000). *The role of social networks in fertility decline in Kenya* (304602204) [Ph.D., Indiana University]. ProQuest Dissertations & Theses Global. http://ezproxy.cul.columbia.edu/login?url=https://search.proquest.com/docview/304602204?accountid=10226

Muvandi, I. (2003). The influence of cultural factors on contraceptive use, fertility attitudes and behaviour in Kenya [Ph.D., University of Surrey (United Kingdom)]. In *PQDT - Global* (1801874388). ProQuest Dissertations & Theses Global. http://ezproxy.cul.columbia.edu/login?url=https://search.proquest.com/docview/1801874388?accountid=10226

Ndiaye, K., Portillo, E., Ouedraogo, D., Mobley, A., & Babalola, S. (2018). High-Risk Advanced Maternal Age and High Parity Pregnancy: Tackling a Neglected Need Through Formative Research and Action. *Global Health Science and Practice*, *6*(2), 370–380. https://doi.org/10.9745/GHSP-D-17-00417

Norris, A. H., Rao, N., Huber-Krum, S., Garver, S., Chemey, E., & Turner, A. N. (2019). Scarcity mindset in reproductive health decision making: A qualitative study from rural Malawi. *Culture Health & Sexuality*, *21*(12), 1333–1348. https://doi.org/10.1080/13691058.2018.1562092

Novignon J., Djossou N.G., & Enemark U. (2019). Childhood mortality, intra-household bargaining power and fertility preferences among women in Ghana. *Reproductive Health*, *16*(1). Embase. https://doi.org/10.1186/s12978-019-0798-2

Nwakeze, N. M. (2007). The demand for children in Anambra State of Nigeria: A logit analysis. *Etude de La Population Africaine*, *22*(2), 167–193. awn.

Nwogu, E. (2006). Family Size Preferences among Women in Anambra State of Nigeria. *Bio-Research*, *4*(2), 148–154. awn.

Nwogu, J. N., Igbolekwu, C. O., Nwokocha, E. E., Nwogu, E. C., Nwabugwu, O. N., & Arisukwu, O. (2021). Roman Catholicism and fertility among the Mbaise, Southeast, Nigeria. *Heliyon*, *7*(2), e05929–e05929. https://doi.org/10.1016/j.heliyon.2021.e05929

Nwokocha, E. E. (2007). Male-Child Syndrome and the Agony of Motherhood Among the Igbo of Nigeria. *International Journal of Sociology of the Family*, *33*(1), 219–234. Sociological Abstracts.

Obembe, T. A., Odebunmi, K. O., & Olalemi, A. D. (2018). Determinants of family size among men in slums of Ibadan, Nigeria. *Annals of Ibadan Postgraduate Medicine*, *16*(1), 12–22. awn.

Odimegwu, O. C. (1994). Motivation as a factor in family size decision-making in a Nigerian ethnic group. *IFE Psychologia: An International Journal*, *2*(1), 123–138. psyh.

Odusina, E. K. (2017). DETERMINANTS OF FERTILITY PREFERENCE AMONG COUPLES IN NIGERIA: IMPLICATION FOR FERTILITY CONTROL. *Gender & Behaviour*, *15*(1), 8406–8420. a9h.

Odusina, E. K., Ayotunde, T., Kunnuji, M., Ononokpono, D. N., Bishwajit, G., & Yaya, S. (2020). Fertility preferences among couples in Nigeria: A cross sectional study. *Reproductive Health*, *17*(1), 92. https://doi.org/10.1186/s12978-020-00940-9

Odusola, A. F. & and others. (1998). Adjustment policies, gender dynamics and family size reduction in Nigeria: A case study of Akwa Ibom state. In *Nigerian Institute of Social and Economic Research (NISER), 1998. 51 pp.* (59799320; 2000-0600210). Nigerian Institute of Social and Economic Research (NISER); PAIS Index. http://ezproxy.cul.columbia.edu/login?url=https://search.proquest.com/docview/59799320?accountid=10226

OGBONNA, D. O., & MAMMAN, M. (1990). Determinants of fertility levels in Kafanchan, Nigeria. *Facteurs Determinants Des Niveaux de Fecondite a Kafanchan, Nigeria.*, *20*(59), 1–23. awn.

Okafor, S., Onu, J. C., & Nwaeze, V. C. (2021). Rural Small-Scale Women Farmers and Preference for Family Size in South-East Nigeria. *Comparative Population Studies*, *46*. https://doi.org/10.12765/CPoS-2021-02

Oladosu, M. (1999). Couple decision making and reproductive outcomes in Uganda [Ph.D., The Pennsylvania State University]. In *ProQuest Dissertations and Theses* (304537586). ProQuest Dissertations & Theses Global. http://ezproxy.cul.columbia.edu/login?url=https://search.proquest.com/docview/304537586?accountid=10226

Olalekan, A. W. (2013). A survey of marriage pattern as determinants of fertility among women in southwestern Nigeria. *TAF Preventive Medicine Bulletin*, *12*(2), 143–150. https://doi.org/10.5455/pmb.1-1331457510

Olaogun A, Ayoola A, Ogunfowokan A, & Ewere V. (2009). Preference for the male child and desired family size in Nigeria. *African Journal of Midwifery & Women’s Health*, *3*(4), 193–197. rzh. https://doi.org/10.12968/ajmw.2009.3.4.44804

Olenick, I. (1998). In Tanzania, Ideal Family Size Closely Resembles Actual Number of Children. *International Family Planning Perspectives*, *24*(3), 147. a9h.

Omariba, D. W. R. (2006). Women’s Educational Attainment and Intergenerational Patterns of Fertility Behaviour in Kenya. *Journal of Biosocial Science*, *38*(4), 449–479. Sociological Abstracts. https://doi.org/10.1017/S0021932005026489

Oshodin, O. G., & Ujiro, I. (2007). Influence of norm of family size of policemen on family planning in Oredo Local Government Area of Edo State, Nigeria. *African Journal for Physical, Health Education, Recreation and Dance*, *13*(4), 465–472.

Osili, U. O., & Long, B. T. (2008). Does female schooling reduce fertility? Evidence from Nigeria. *Journal of Development Economics*, *87*(1), 57–75. https://doi.org/10.1016/j.jdeveco.2007.10.003

Owoo, N. S., Agyei-Mensah, S., & Onuoha, E. (2015). The effect of neighbourhood mortality shocks on fertility preferences: A spatial econometric approach. *The European Journal of Health Economics*, *16*(6), 629–645. psyh. https://doi.org/10.1007/s10198-014-0615-3

Owoo, N. S., & Lambon-Quayefio, M. P. (2021). Does Job Security Affect Fertility and Fertility Intentions in Ghana? Examining the Evidence. *Journal of Family and Economic Issues*. https://doi.org/10.1007/s10834-021-09758-4

Oyediran, K. A. (2006). Fertility desires of Yoruba couples of South-western Nigeria. *Journal of Biosocial Science*, *38*(5), 605–624. https://doi.org/10.1017/S0021932004026835

Oyediran, K. A., & Isiugo-Abanihe, U. C. (2002). Husband-Wife Communication and Couple’s Fertility Desires Among the Yoruba of Nigeria. *Etude de La Population Africaine*, *17*(2), 61–80. awn.

Perkins, A. C. J. (1997). Fertility and household bargaining in sub-Saharan Africa: How large is the husband’s influence? [Ph.D., Cornell University]. In *ProQuest Dissertations and Theses* (304386151). ProQuest Dissertations & Theses Global. http://ezproxy.cul.columbia.edu/login?url=https://search.proquest.com/docview/304386151?accountid=10226

Pierotti, R. S. (2013). Gender Ideologies: Insights into Health and Demographic Behaviors [Ph.D., University of Michigan]. In *ProQuest Dissertations and Theses* (1497967320). Sociological Abstracts. http://ezproxy.cul.columbia.edu/login?url=https://search.proquest.com/docview/1497967320?accountid=10226

Poukouta, P. V., & Fapohunda, B. M. (1997). Trends and Differentials in Desired Family Size in Kenya. *Etude de La Population Africaine*, *12*(1). awn. https://proxy.lib.ohio-state.edu/login?url=http://search.ebscohost.com/login.aspx?direct=true&db=awn&AN=1419281&site=ehost-live

Price, N. (1995a). The social and institutional context of high fertility amongst the Gwembe Valley Tonga of Zambia. *Papers in International Development - Centre for Development Studies, University College of Swansea*, *11*, 27-p.

Price, N. (1995b). The value of children amongst the Kikuyu of Central Province, Kenya: An anthropological perspective on fertility. *Papers in International Development - Centre for Development Studies, University College of Swansea*, *13*(13), 28-p.

Randall, S., & LeGrand, T. (2003). Reproductive Strategies and Decisions in Senegal: The Role of Child Mortality. *Population*, *58*(6), 687–715. International Bibliography of the Social Sciences (IBSS). https://doi.org/10.2307/3246671

Ratcliffe, A. A., Hill, A. G., & Walraven, G. (2000). Separate lives, different interests: Male and female reproduction in the Gambia. *Bulletin of the World Health Organization*, *78*(5), 570–579. International Bibliography of the Social Sciences (IBSS).

Reynar, A. R. (2000). Fertility decision -making by couples amongst the Luo of Kenya [Ph.D., University of Pennsylvania]. In *ProQuest Dissertations and Theses* (304615595). ProQuest Dissertations & Theses Global. http://ezproxy.cul.columbia.edu/login?url=https://search.proquest.com/docview/304615595?accountid=10226

Robilliard, A.-S. (2020). Empowerment and Desired Fertility in Sub Saharan Africa. *Advances in Gender Research*, *29*, 39–64. https://doi.org/10.1108/S1529-212620200000029002

Rutayisire, P. C., Broekhuis, A., & Hooimeijer, P. (2013). Role of conflict in shaping fertility preferences in Rwanda. *African Population Studies*, *27*(2), 105–117. sih. https://doi.org/10.11564/27-2-433

Sahleyesus, D. T., Beaujot, R. P., & Zakus, D. (2009). Attitudes toward family size preferences in urban Ethiopia. *Journal of Comparative Family Studies*, *40*(1), 97–117. psyh.

Saila-Ngita, D., Bravo-Ureta, B. E., & Perez-Escamilla, R. (2003). Fertility desires and sample selection bias: The case of Senegal. *Journal of Asian and African Studies*, *38*(1), 1–16. International Bibliography of the Social Sciences (IBSS). https://doi.org/10.1177/002190960303800101

Sam, D. L., Peltzer, K., & Mayer, B. (2005). The Changing Values of Children and Preferences Regarding Family Size in South Africa. *Applied Psychology: An International Review*, *54*(3), 355–377. psyh. https://doi.org/10.1111/j.1464-0597.2005.00215.x

Samandari, G., Grant, C., Brent, L., & Gullo, S. (2019). “It is a thing that depends on God”: Barriers to delaying first birth and pursuing alternative futures among newly married adolescent girls in Niger. *Reproductive Health*, *16*(1), 109. https://doi.org/10.1186/s12978-019-0757-y

Sathiya Susuman A., Bado A., & Lailulo Y.A. (2014). Promoting family planning use after childbirth and desire to limit childbearing in Ethiopia. *Reproductive Health*, *11*(1), 53. Embase. https://doi.org/10.1186/1742-4755-11-53

Shapiro, D. (2018). *Emerging Preferences for Low Fertility in Sub-Saharan Africa-Aug. 2018*.

Shapiro, D., & Tenikue, M. (2017). Women’s education, infant and child mortality, and fertility decline in rural and urban sub-Saharan Africa. *Demographic Research*, *37*, 669–708. https://doi.org/10.4054/DemRes.2017.37.21

Short, S. E., & Kiros, G.-E. (2002). Husbands, wives, sons, and daughters: Fertility perferences and the demand for contraception in Ethiopia. *Population Research and Policy Review*, *21*(5), 377–402. PAIS Index. https://doi.org/10.1023/A:1021185812984

Shreffler, K. M., & Dodoo, F. N.-A. (2009). The role of intergenerational transfers, land, and education in fertility transition in rural Kenya: The case of Nyeri district. *Population & Environment*, *30*(3), 75–92. a9h. https://doi.org/10.1007/s11111-009-0077-1

Sinai, I., Omoluabi, E., Jimoh, A., & Jurczynska, K. (2019). Unmet need for family planning and barriers to contraceptive use in Kaduna, Nigeria: Culture, myths and perceptions. *Culture, Health & Sexuality*, 1–16. https://doi.org/10.1080/13691058.2019.1672894

Smith, D. J. (1999). Having people: Fertility, family and modernity in Igbo-speaking Nigeria [Ph.D., Emory University]. In *ProQuest Dissertations and Theses* (304553755). ProQuest Dissertations & Theses Global. http://ezproxy.cul.columbia.edu/login?url=https://search.proquest.com/docview/304553755?accountid=10226

Smith, D. J. (2004). Contradictions in Nigeria’s fertility transition: The burdens and benefits of having people. *Population and Development Review*, *30*(2), 221–238. Scopus. https://doi.org/10.1111/j.1728-4457.2004.011_1.x

Snow, R. C., Winter, R. A., & Harlow, S. D. (2013). Gender Attitudes and Fertility Aspirations among Young Men in Five High Fertility East African Countries. *Studies in Family Planning*, *44*(1), 1–24. https://doi.org/10.1111/j.1728-4465.2013.00341.x

Solanke, B. L. (2017). Advanced reproductive age and childbearing choices in Nigeria. *Health Care for Women International*, *38*(6), 640–657. rzh. https://doi.org/10.1080/07399332.2017.1297449

Speizer, I. (1995). Men’s desire for additional wives and children. *Biodemography and Social Biology*, *42*(3–4), 199–213. psyh. https://doi.org/10.1080/19485565.1995.9988901

Speizer, I. (1999). Men, marriage, and ideal family size in Francophone Africa. *Journal of Comparative Family Studies*, *30*(1), 17–34. ssa. https://doi.org/10.3138/jcfs.30.1.17

Speizer, I. S. (1995). A marriage trichotomy and its applications. *Demography*, *32*(4), 533–542. Scopus. https://doi.org/10.2307/2061673

Spjeldnaes, I. O., Sam, D. L., Moland, K. M., & Peltzer, K. (2007). Continuity and change in reproductive attitudes of teenage women, their mothers, and maternal grandmothers in South Africa. *South African Journal of Psychology*, *37*(4), 856–877. https://doi.org/10.1177/008124630703700412

Staveteig, S. E. (2011). Genocide, Nuptiality, and Fertility in Rwanda and Bosnia-Herzegovina [Ph.D., University of California, Berkeley]. In *ProQuest Dissertations and Theses* (928955629). ProQuest Dissertations & Theses Global. http://ezproxy.cul.columbia.edu/login?url=https://search.proquest.com/docview/928955629?accountid=10226

Suda, C. A. (1997). Fertility and the status of women in Kericho District: Reflections on some key reproductive issues. *Kenya Journal of Sciences. Series C, Humanities and Social Sciences*, *4*(1), 61–76. awn.

Swartz, A., Colvin, C., & Harrison, A. (2018). The problem or the solution? Early fertility and parenthood in the transition to adulthood in Khayelitsha, South Africa. *Reproductive Health Matters*, *26*(54), 145–154. a9h. https://doi.org/10.1080/09688080.2018.1537417

Tadesse, B., & Asefa, S. (2002). Empirical analysis of the determinants of demand for children in Jimma city, Ethiopia: An application of count data model. *Eastern Africa Social Science Research Review*, *18*(2), 43–67. awn.

Tadesse, F., & Headey, D. (2010). Urbanization and fertility rates in Ethiopia. *Ethiopian Journal of Economics*, *19*(2), 35–72. awn.

Takyi, B. K. (1993). *The status of women and fertility behavior in sub-Saharan Africa: The effects of female labor force participation and gender preferences on fertility in Ghana* (304091015) [Ph.D., State University of New York at Albany]. ProQuest Dissertations & Theses Global. http://ezproxy.cul.columbia.edu/login?url=https://search.proquest.com/docview/304091015?accountid=10226

Telake, D. S. (2006). Fertility transition in Ethiopia: A focus on urban fertility and the urban-rural differences [Ph.D., The University of Western Ontario (Canada)]. In *ProQuest Dissertations and Theses* (304941233). ProQuest Dissertations & Theses Global. http://ezproxy.cul.columbia.edu/login?url=https://search.proquest.com/docview/304941233?accountid=10226

Teye, J. K. (2013). Economic value of children and fertility preferences in a fishing community in Ghana. *GeoJournal*, *78*(4), 697–708. International Bibliography of the Social Sciences (IBSS). https://doi.org/10.1007/s10708-012-9460-z

Thiede, B. C., Hancock, M., Kodouda, A., & Piazza, J. (2020). Exposure to Armed Conflict and Fertility in Sub-Saharan Africa. *Demography*, *57*(6), 2113–2141. https://doi.org/10.1007/s13524-020-00923-2

Thomas, J. (2013). Young Men Who Feel That Wife Beating Is Sometimes Acceptable Have Elevated Fertility Desires. *International Perspectives on Sexual & Reproductive Health*, *39*(2), 106–106. a9h.

Togunde, D., & Newman, S. (2005). Value of children, child labor, and fertility preferences in urban Nigeria. *West Africa Review*, *7*. PAIS Index. http://ezproxy.cul.columbia.edu/login?url=https://search.proquest.com/docview/59947804?accountid=10226

Towriss, C. A. (2014). Birth intervals and reproductive intentions in eastern africa: Insights from urban fertility transitions [Ph.D., University of London, London School of Hygiene and Tropical Medicine (United Kingdom)]. In *PQDT - UK & Ireland* (1779253745). ProQuest Dissertations & Theses Global. http://ezproxy.cul.columbia.edu/login?url=https://search.proquest.com/docview/1779253745?accountid=10226

Towriss, C. A., Beguy, D., Wringe, A., Hussein, B. H., & Timæus, I. M. (2020). Planning a family in Nairobi’s informal settlements: Results of a qualitative study. *Journal of Biosocial Science*, *52*(2), 286–299. https://doi.org/10.1017/S0021932019000452

Trinitapoli, J., & Yeatman, S. (2011). Uncertainty and Fertility in a Generalized AIDS Epidemic. *American Sociological Review*, *76*(6), 935–954. a9h. https://doi.org/10.1177/0003122411427672

Trinitapoli, J., & Yeatman, S. (2018). The flexibility of fertility preferences in a context of uncertainty. *Population and Development Review*, *44*(1), 87–116. https://doi.org/10.1111/padr.12114

Uchudi, J. M. (1999). Gender context and fertility behavior in sub-Saharan Africa [Ph.D., Cornell University]. In *ProQuest Dissertations and Theses* (304503236). ProQuest Dissertations & Theses Global. http://ezproxy.cul.columbia.edu/login?url=https://search.proquest.com/docview/304503236?accountid=10226

Ukaegbu, A. O. (2013). Family size preferences of spouses in Rural Eastern Nigeria. In *Population and Development: High and Low Fertility in Poorer Countries* (Vol. 20, pp. 150–164). Scopus. https://www.scopus.com/inward/record.uri?eid=2-s2.0-85070430311&partnerID=40&md5=63ebdb9805905985697bf21dec47d412

Umoh, A. V., Abah, G. M., & Ekanem, U. S. (2012). A study of fertility intentions of women in Uyo, Nigeria. *Journal of Public Health and Epidemiology*, *4*(1), 14–18.

Upadhyay, U. D., & Karasek, D. (2012). Women’s Empowerment and Ideal Family Size: An Examination of DHS Empowerment Measures In Sub-Saharan Africa. *International Perspectives on Sexual & Reproductive Health*, *38*(2), 78–89. rzh. https://doi.org/10.1363/3807812

Uzobo, E., & Odubo, T. R. (2016). Economic Empowerment of Women and Fertility Behaviour in Ogbia Local Government Area, Bayelsa State, Nigeria. *African Research Review*, *10*(2), 64–80. awn.

Van de Walle F. & Maiga M. (1991). Family planning in Bamako, Mali. *International Family Planning Perspectives*, *17*(3), 84-90+99. Embase.

Vohra, D. (2014). *Understanding the Gap Between Fertility Intentions and Outcomes* (1666860457) [Ph.D., University of California, Berkeley]. ProQuest Dissertations & Theses Global. http://ezproxy.cul.columbia.edu/login?url=https://search.proquest.com/docview/1666860457?accountid=10226

Westoff, C. F., & Bietsch, K. (2015). Religion and reproductive behavior in sub-Saharan Africa. *DHS Analytical Studies*, *48*, xi + 26 pp.

Westoff, C. F., & Rodriguez, G. (1995). The mass media and family planning in Kenya. *International Family Planning Perspectives*, *21*(1), 26–31, 36. https://doi.org/10.2307/2133602

Wilson, J. F. (1998). Reproductive decision making among Zambian couples: Agreement and conflict [M.S., University of North Texas]. In *ProQuest Dissertations and Theses* (304448083). ProQuest Dissertations & Theses Global. http://ezproxy.cul.columbia.edu/login?url=https://search.proquest.com/docview/304448083?accountid=10226

Woldemicael, G. (2009). Womens autonomy and reproductive preferences in Eritrea. *Journal of Biosocial Science*, *41*(2), 161–181. Scopus. https://doi.org/10.1017/S0021932008003040

Woldesenbet, A. K. (2010). *Determinants of fertility decisions at a household level in rural Ethiopia [Language: Eng]* (Location: University Library, Norwegian Univ. of Life Sciences, P.O.Box 5003, N-1432 Aas - Norway. E-mail biblutl@umb.no; Number: 112721907). 68–68. awn.

Yeatman, S., & Sennott, C. (2014). The Relationship between Partners’ Family-Size Preferences in Southern Malawi. *Studies in Family Planning*, *45*(3), 361–377. a9h. https://doi.org/10.1111/j.1728-4465.2014.00396.x

Yeatman, S., Sennott, C., & Culpepper, S. (2013). Young women’s dynamic family size preferences in the context of transitioning fertility. *Demography*, *50*(5), 1715–1737. rzh. https://doi.org/10.1007/s13524-013-0214-4

1. No search terms or limits pertaining to the population (i.e. sex and age) were included during the search to avoid inadvertently eliminating relevant results. [↑](#endnote-ref-1)
